# Author Correction: Genetic regulation of *TERT* splicing affects cancer risk by altering cellular longevity and replicative potential

**DOI:** 10.1038/s41467-025-63402-5

**Published:** 2025-08-28

**Authors:** Oscar Florez-Vargas, Michelle Ho, Maxwell H. Hogshead, Brenen W. Papenberg, Chia-Han Lee, Kaitlin Forsythe, Kristine Jones, Wen Luo, Kedest Teshome, Cornelis Blauwendraat, Kimberley J. Billingsley, Mikhail Kolmogorov, Melissa Meredith, Benedict Paten, Raj Chari, Chi Zhang, John S. Schneekloth, Mitchell J. Machiela, Stephen J. Chanock, Shahinaz M. Gadalla, Sharon A. Savage, Sam M. Mbulaiteye, Ludmila Prokunina-Olsson

**Affiliations:** 1 Laboratory of Translational Genomics, DCEG, National Cancer Institute, Rockville, MD USA; 2 Cancer Genomics Research Laboratory, Leidos Biomedical Research, Frederick National Laboratory for Cancer Research, Frederick, MD USA; 3 Center for Alzheimer’s and Related Dementias, National Institute of Aging and National Institute of Neurological Disorders and Stroke, Bethesda, MD USA; 4 Cancer Data Science Laboratory, CCR, National Cancer Institute, Bethesda, MD USA; 5 UC Santa Cruz Genomics Institute, Santa Cruz, CA USA; 6 Genome Modification Core, Laboratory Animal Sciences Program, Leidos Biomedical Research, Frederick National Laboratory for Cancer Research, Frederick, MD USA; 7 Chemical Biology Laboratory, CCR, National Cancer Institute, Frederick, MD USA; 8 Integrative Tumor Epidemiology Branch, DCEG, National Cancer Institute, Rockville, MD USA; 9 Laboratory of Genetic Susceptibility, DCEG, National Cancer Institute, Rockville, MD USA; 10 Clinical Genetics Branch, DCEG, National Cancer Institute, Rockville, MD USA; 11 Infections and Immunoepidemiology Branch, DCEG, National Cancer Institute, Rockville, MD USA

**Keywords:** Cancer genetics, Gene regulation, Genetic association study

Correction to: *Nature Communications* 10.1038/s41467-025-56947-y, published online 16 February 2025

In this article the author name Kimberley J. Billingsley was incorrectly written as Kimberly J. Billingsley. The original article has been corrected.

