# Supplementary material for: Genetic regulation of TERT splicing affects cancer risk by altering cellular longevity and replicative potential
Source: Nat Commun. 2025 Feb 16;16:1676. doi: 10.1038/s41467-025-56947-y (PMC11830802; doi:10.1038/s41467-025-56947-y)
Supplement: Supplementary file 1 — Supplementary Information [file 41467_2025_56947_MOESM1_ESM.pdf]

Supplementary Figures

Genetic regulation of *TERT* splicing affects cancer risk by altering cellular longevity and replicative potential

Florez-Vargas et al.

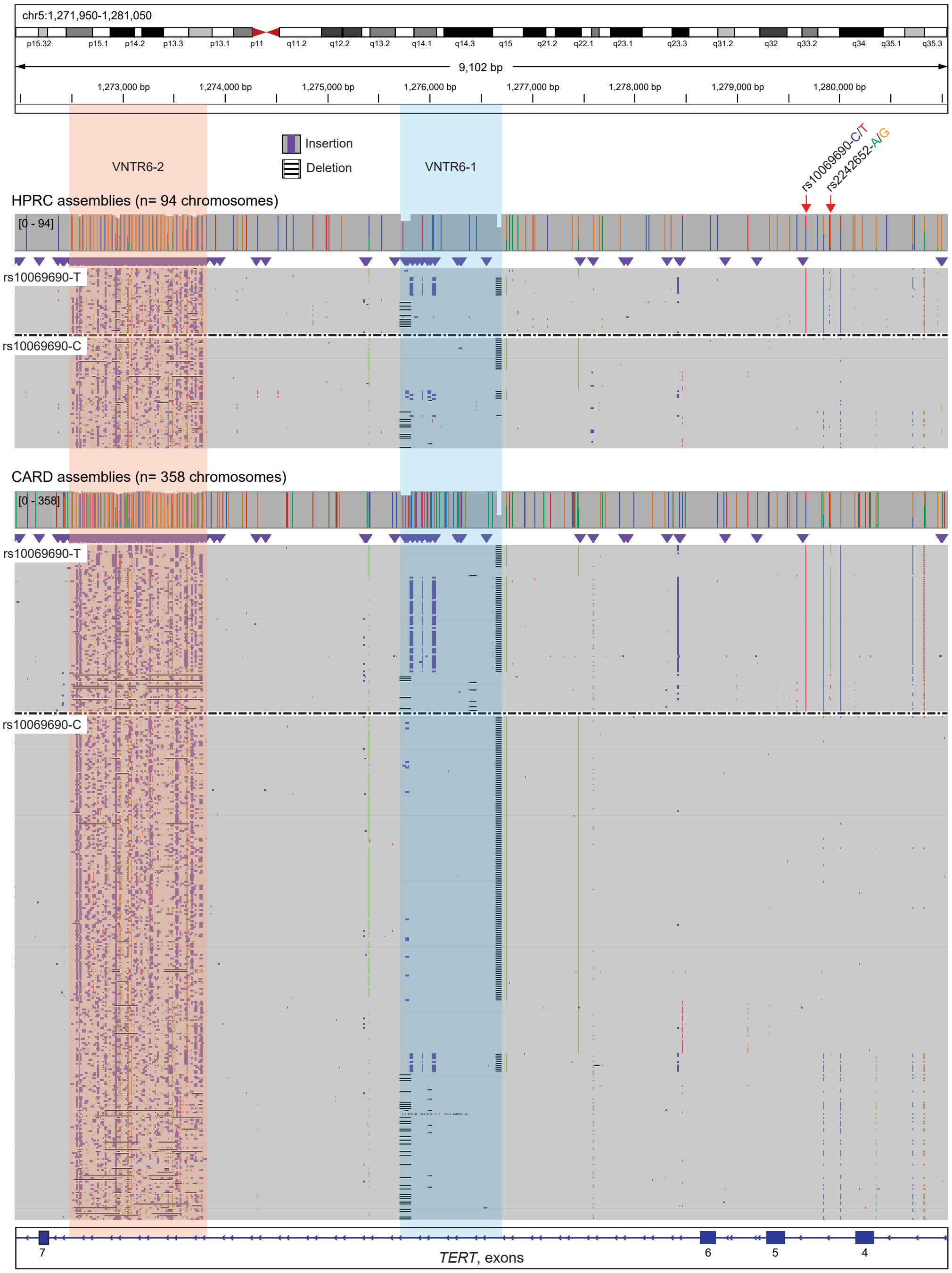

Supplementary Figure 1. Analysis of *TERT* VNTR6-1 and VNTR6-2 in long-read WGS assemblies.

Integrative Genomics Viewer (IGV) plots of long-read genome assemblies (n=452) for HPRC and CARD participants. The region of interest (GRCh38 chr5:1,250,000-1,450,000) features VNTR6-1 (blue highlight) and VNTR6-2 (pink highlight) within intron 6 and multi-cancer GWAS signals rs10069690 and rs2242652 within intron 4 (gray – base matches, colored – mismatches with the reference genome). Each individual is represented by two assemblies (rows). Insertions (purple marks) in the VNTR6-1 region are enriched in the assemblies with rs10069690-T and rs2242652-A alleles. Compared with the alternative alleles, assemblies with the rs2242652-A allele have more copies of VNTR6-1 (p=5.93E-19) and VNTR6-2 (p=7.66E-04). Assemblies with the rs10069690-T allele have more copies of VNTR6-1 (p=5.40E-11) but not VNTR6-2 (p=0.84). Gray marks within the VNTR6-1 region correspond to the deletion of 53 bp (~1.5 repeats) segregating with 25.5, 40.5 and 66.5 copies (alleles) of VNTR6-1 but independent of rs10069690 and rs2242652. The details and source data are provided in the Source Data file.

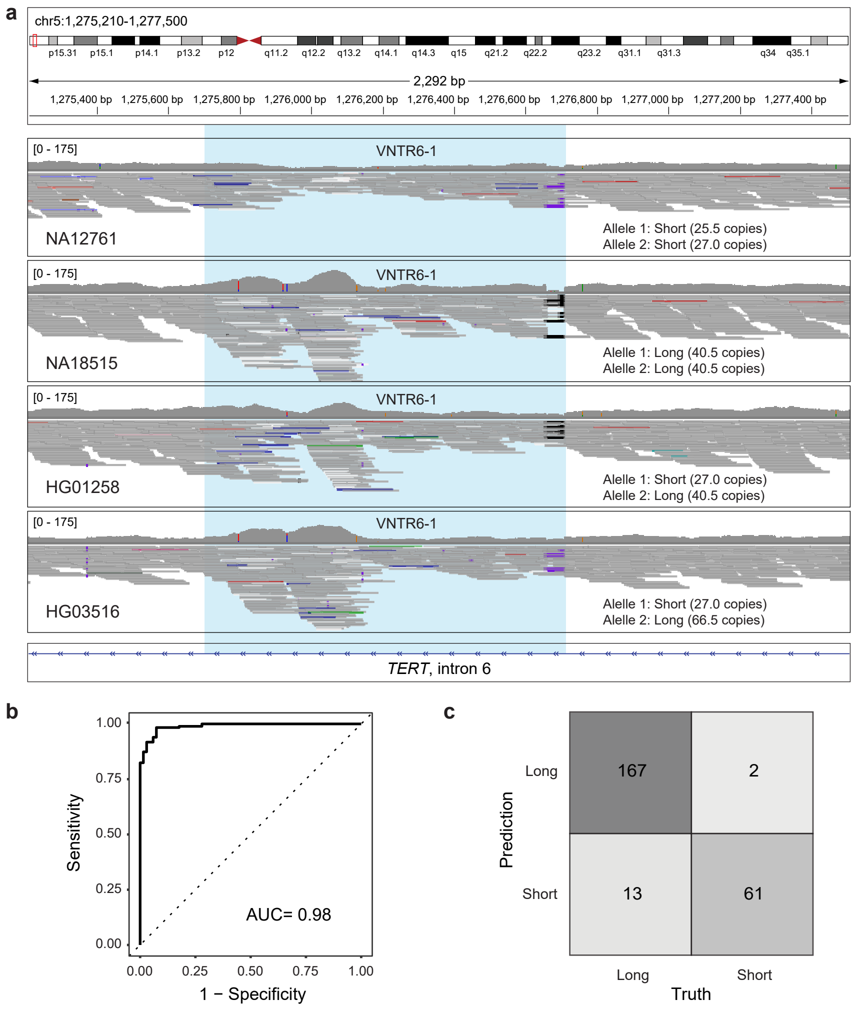

Supplementary Figure 2. Analysis of VNTR6-1 in short-read WGS alignments in the 1000G samples.

a, Representative IGV plots of short-read WGS alignments (Illumina, 30x coverage) illustrating the VNTR6-1 genomic profiles (blue highlight) of four 1000G samples. The VNTR6-1 repeat copies were determined by targeted PacBio sequencing and long-read HPRC assemblies, assigning Short/Short genotype (25.5 and 27 copies) for NA12761, Long/Long genotype (homozygous for 40.5 copies) for NA18515, Short/Long genotype (27.0 and 40.0 copies) for HG01258, and Short/Long genotype (27.0 and 66.5 copies) for HG03516. The short-read WGS profiles distinguish only between VNTR6-1 Short/Short vs. Long/Any genotypes but not within these groups. **b**, The performance of the machine learning binary classification model, which categorizes VNTR6-1 into Short and Long groups based on short-read WGS profiles using regularized multimodal logistic regression assessed in 605 samples (18.9% of the dataset) randomly selected to represent all 1000G super-populations. Samples were visually classified as Short or Long based on IGV coverage profiles. The dataset was split into training (60%, n=362) and testing (40%, n=243) subsets, with fivefold cross-validation during training. In the testing set, the model demonstrated robust accuracy, achieving 96.8% specificity, 92.8% sensitivity, and an F score of 0.95, effectively distinguishing between VNTR6-1 Long/any and Short/Short genotype groups, as illustrated in the receiver operating characteristic (ROC) curve (AUC=0.98) and **c**, confusion matrix evaluation. The source data are provided in the Source Data file.

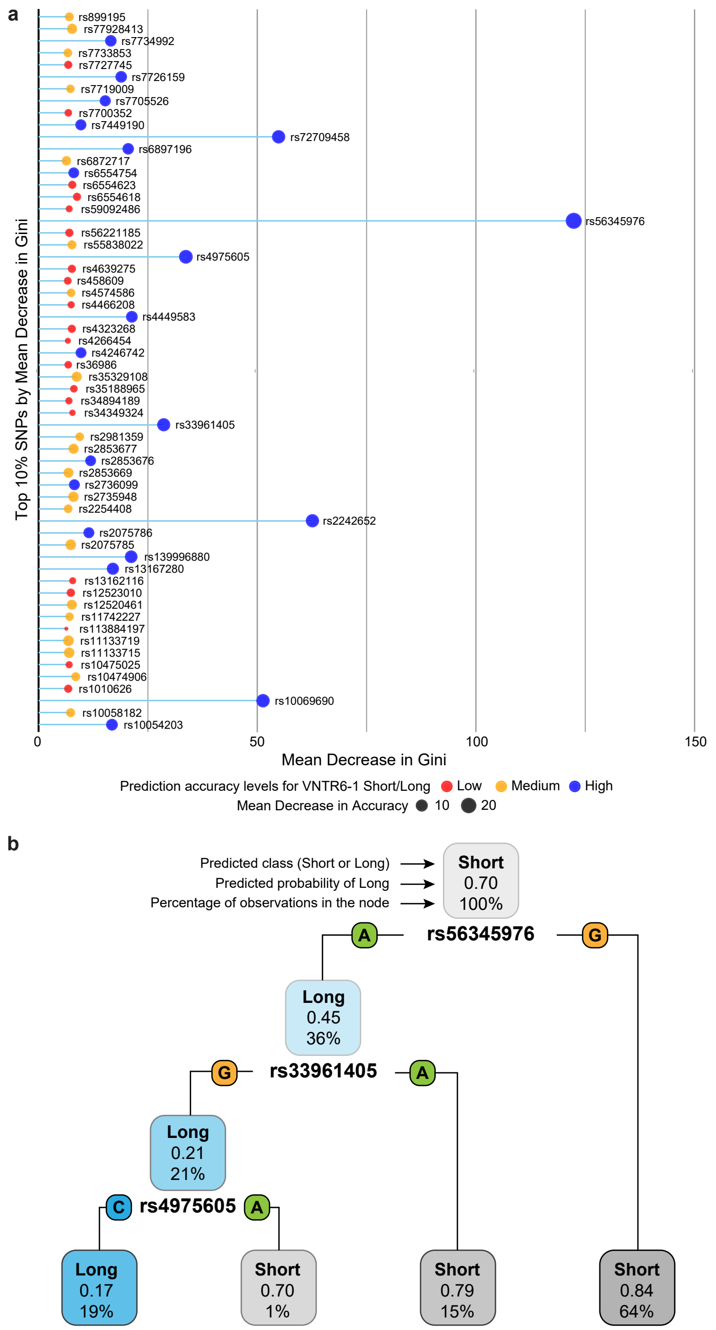

Supplementary Figure 3. Random forest machine learning analysis of SNP-based prediction of VNTR6-1 groups in the 1000G samples.

a, Starting from 1,473 SNPs with MAF>5% within the 400 kb genomic region (GRCh38 chr5:1,100,000-1,500,000), 594 SNPs were significantly associated with the VNTR6-1 Short and Long categories ($p<0.05$, Chi-squared test), which were established for each of the 1000G samples (n=3201) based on short-read WGS profiles. The top 10% of those SNPs were selected based on a Mean Decrease in Gini values, indicating their high discriminative power to predict VNTR6-1 Short/Short and Long/any genotype groups. Dot sizes represent a Mean Decrease in Accuracy, with larger circles corresponding to higher model accuracy and higher values corresponding to substantial accuracy loss if the feature is removed. SNPs with both a high Mean Decrease in Gini and a high Mean Decrease in Accuracy are the most informative for distinguishing the VNTR6-1 groups. **b**, A representative decision tree from the ensemble of 500 trees in the random forest model illustrates the classification of VNTR6-1 groups. Each node in the tree represents a decision point based on the values of the predictor variables, leading to sample assignment either to VNTR6-1 Short/Short or Long/any groups. The source data are provided in the Source Data file.

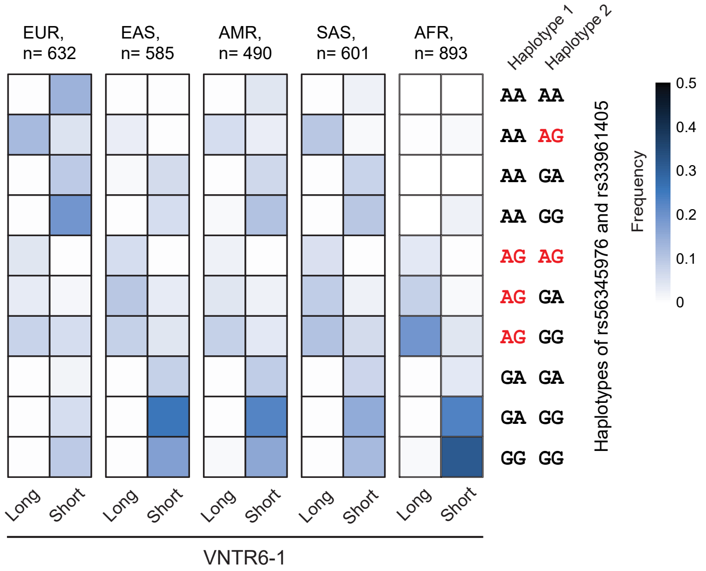

Supplementary Figure 4. Clustering of rs56345976/rs33961405 haplotypes with VNTR6-1 groups across the 1000G super-populations.

Analysis of the 3,201 individuals from the 1000G super-populations shows clustering of the rs56345976-A/rs33961405-G haplotype with the VNTR6-1-Long group determined by short-read WGS profiles, whereas all other haplotypes cluster with the VNTR6-1-Short group. The source data are provided in the Source Data file.

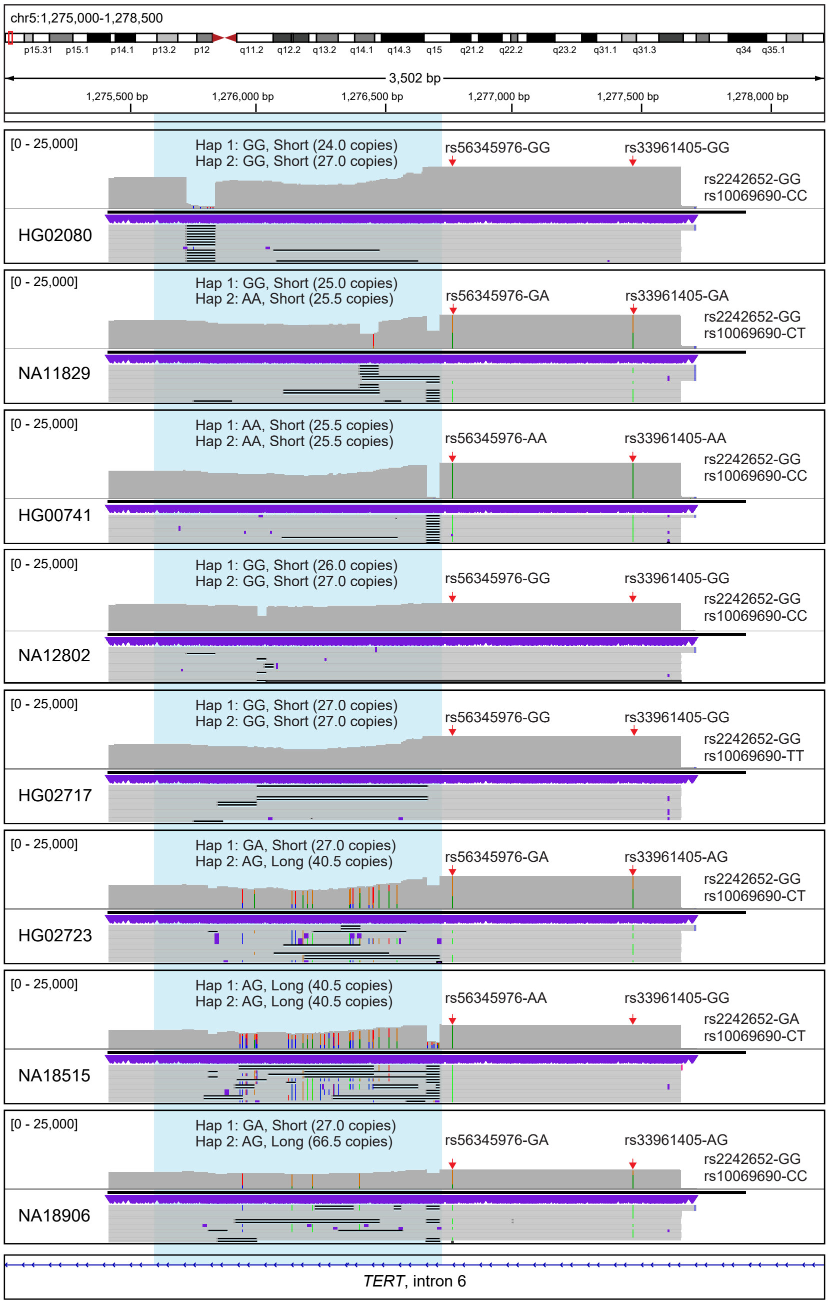

Supplementary Figure 5. VNTR6-1 and rs56345976/rs33961405 in targeted long-read PacBio sequencing alignments.

Representative IGV plots for targeted PacBio sequencing alignments for HapMap/1000G samples illustrating VNTR6-1 (blue highlight) and SNPs rs56345976 and rs33961405 (red arrows). Hap 1 and Hap 2 present phased haplotypes of rs56345976 and rs33961405 alleles and corresponding VNTR6-1 repeat copies determined based on Tandem Repeat Finder analysis of PacBio sequencing reads and HPRC long-read genome assemblies (where available). The select samples demonstrate the range of VNTR6-1 repeat sizes observed in human populations (24-66.5 copies). The genotypes of the multi-cancer GWAS leads rs2242652 and rs10069690 located outside of the sequenced amplicon are shown for corresponding samples. The source data are provided in the Source Data file.

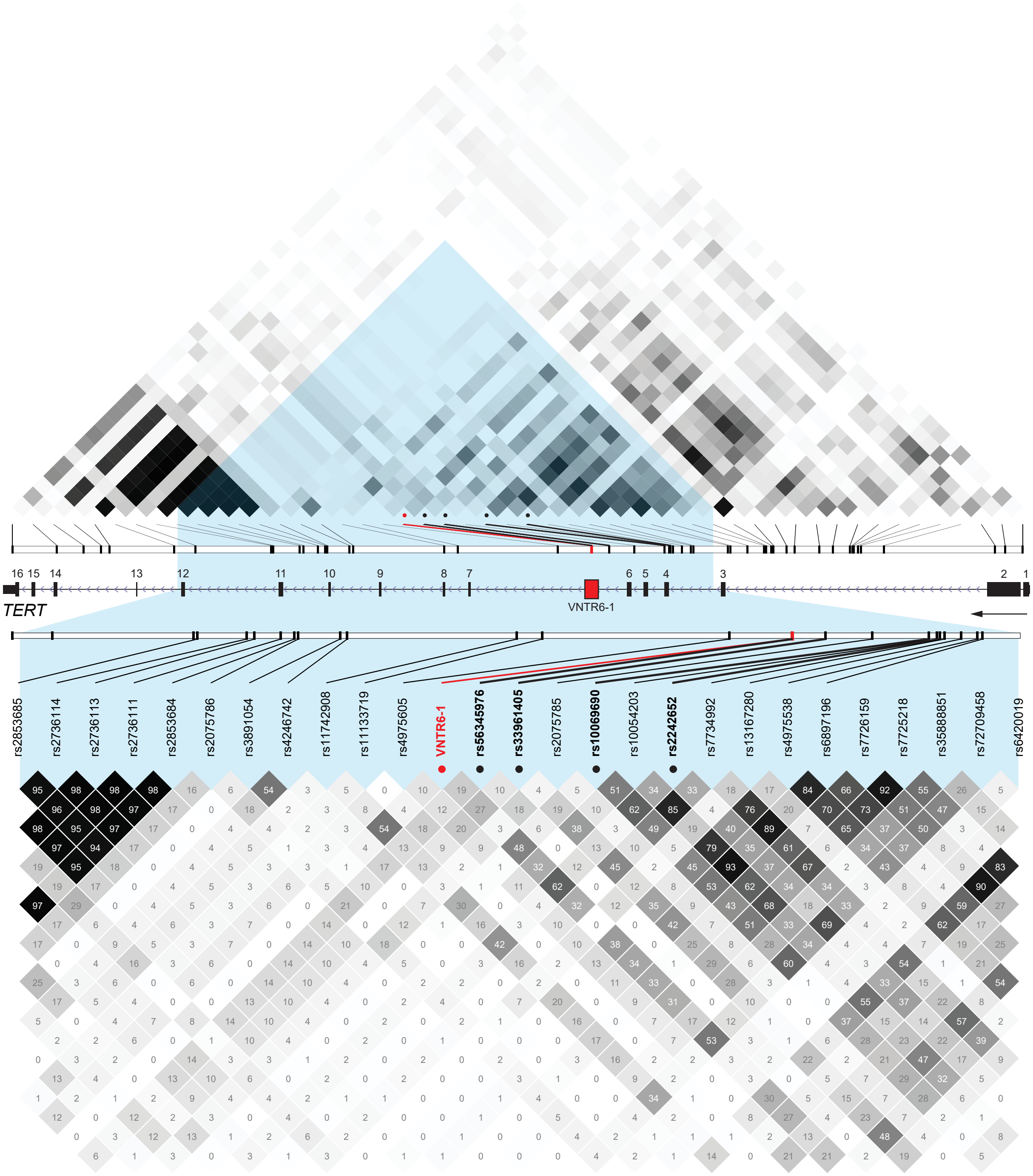

Supplementary Figure 6. Linkage disequilibrium (LD) profile of the *TERT* region in the custom reference panel of 1000G-EUR samples.

The custom reference panel for 632 individuals of European ancestry from 1000G populations was constructed for the 400 kb genomic region (GRCh38, chr5:1,100,000-1,500,000) by adding VNTR6-1 as a biallelic marker (Short and Long alleles) to the existing high-coverage (30x) WGS genotype data downloaded from https://www.internationalgenome.org/data-portal/data-collection/30x-grch38 (shown at MAF>0.05, HWE p>0.001). The upper panel shows the whole *TERT* region, and the lower panel details the area with the relevant markers – VNTR6-1, which is constructed based on rs56345976/rs33961405 haplotypes, and GWAS signals in intron 4 – rs10069690 and rs2242652. Darker shading on the LD plots corresponds to higher r^2 between markers, also shown as values in boxes. The source data are provided in the Source Data file.

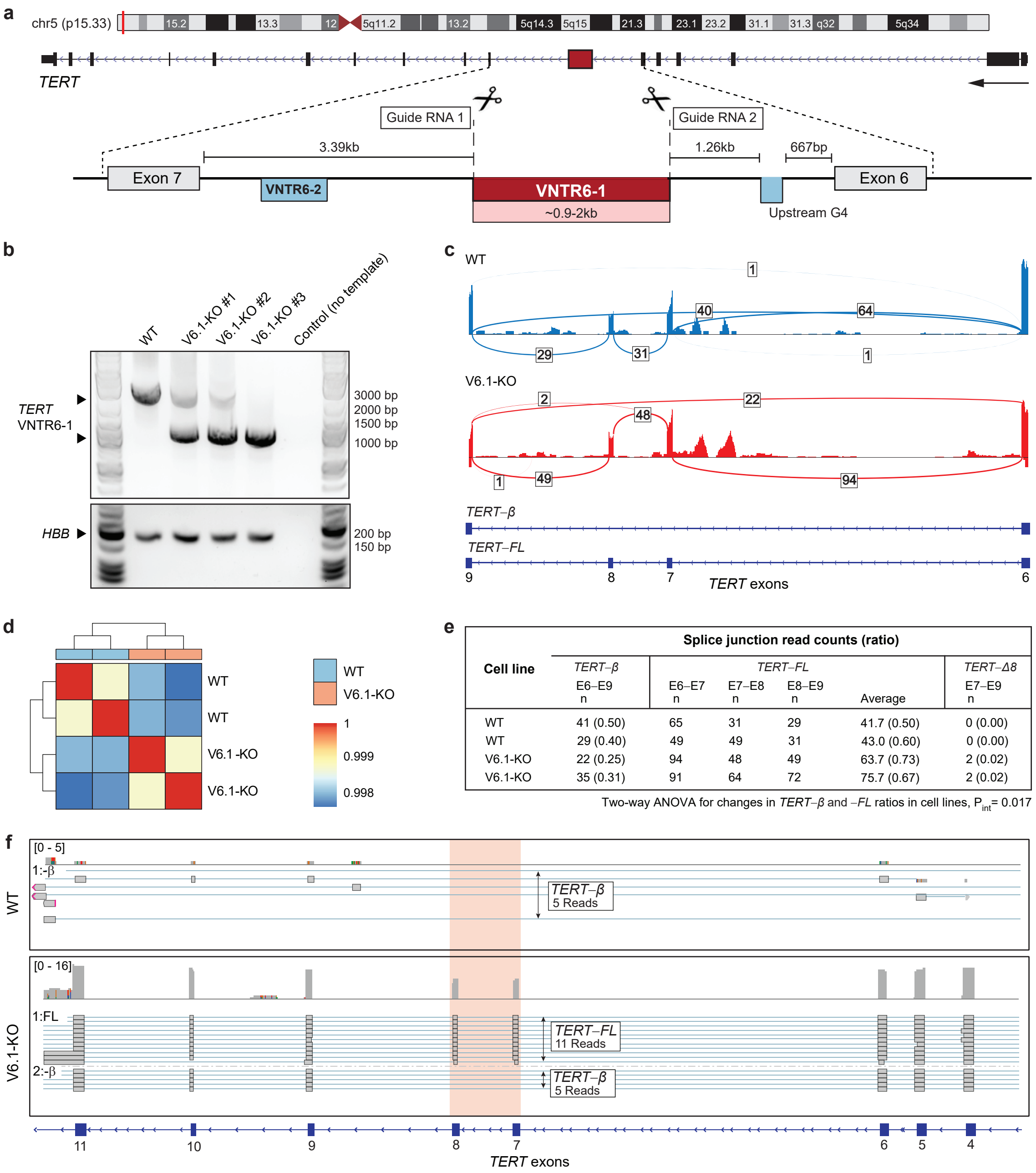

Cell line	TERT–β E6–E9 n	TERT–FL E6–E7 n	TERT–FL E7–E8 n	TERT–FL E8–E9 n	TERT–FL Average	TERT–Δ8 E7–E9 n
WT	41 (0.50)	65	31	29	41.7 (0.50)	0 (0.00)
WT	29 (0.40)	49	49	31	43.0 (0.60)	0 (0.00)
V6.1-KO	22 (0.25)	94	48	49	63.7 (0.73)	2 (0.02)
V6.1-KO	35 (0.31)	91	64	72	75.7 (0.67)	2 (0.02)

Supplementary Figure 7. Splicing effects of *TERT*-VNTR6-1 knockout in UMUC3 cells.

a, CRISPR/Cas9 gene editing strategy for creating V6.1-KO cell lines. The 20 nt protospacers of CRISPR/Cas9 guide RNAs were placed 133 bp upstream and 82 bp downstream of VNTR6-1 within intron 6 of *TERT*. **b**, Agarose gels of PCR products amplified from genomic DNA of UMUC3, a bladder cancer cell line; *HBB* amplicon - normalization control. **c,** Representative IGV-Sashimi plots showing RNA-seq splicing profiles of WT and V6.1-KO cell lines. **d,** A heatmap showing similar clustering of biological RNA-seq duplicates of WT and V6.1-KO. **e,** RNA-seq splicing profiles based on biological duplicates of WT and V6.1-KO cell lines (including one from plot **c**). V6.1-KO causes a significant change in *TERT* splicing pattern (ANOVA p=0.017), with an increase in *TERT-FL* and a decrease in *TERT-β* isoform expression. **f**, IGV coverage map for Oxford Nanopore cDNA sequencing of WT and V6.1-KO cell lines; the highlighted region between *TERT* exons 7 and 8 shows the position of *TERT-FL* and *TERT-β*, with an increase of *TERT-FL* in V6.1-KO cell line, similarly to what was observed by short-read RNA-seq (panels **c-e**). The source data are provided in the Source Data file.

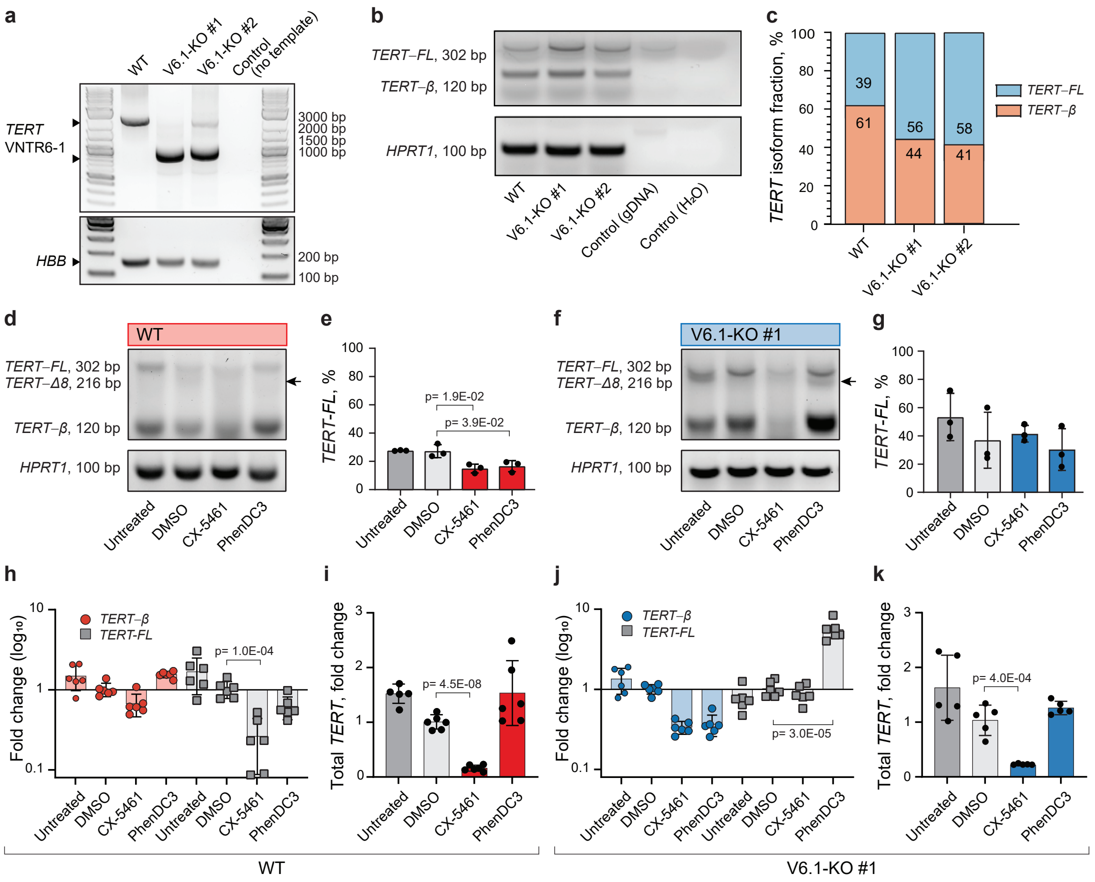

Supplementary Figure 8. Splicing effects of *TERT*-VNTR6-1 knockout in A549 cells.

a, Agarose gels of PCR products amplified from genomic DNA of A549, a lung cancer cell line; *HBB* amplicon - normalization control. **b**, Agarose gels of RT–PCR products amplified from cDNA of corresponding samples; gDNA–genomic DNA, negative control; *HPRT1* - normalization control. **c**, Densitometry results of the PCR amplicons in plot **b**. Experiments in A549 cells comparing *TERT* splicing and isoform-specific expression after 72 hours of treatment with G4-stabilizing ligands, normalized to *HPRT1* as an endogenous control in the WT **d**, **e** and V6.1-KO **f**, **g** cell lines. **d**, **f**, A representative agarose gel of SYBR-Green RT–qPCR products detecting several isoforms with primers located in exons 6 and 9. The extra PCR band, marked by an arrow in panels **c** and **d**, is further explored in **Supplementary Figure 12**. **e**, **g**, Densitometry analysis of the corresponding agarose gels evaluating the percentage of *TERT-FL* (%) relative to the total PCR products. **h**, **j**, Isoform-specific TaqMan RT-qPCR analysis of *TERT-FL* and *TERT-β* following treatment with G4-stabilizing ligands CX-5461 and PhenDC3 for 72 hours. **i**, **k**, Total *TERT* expression measured by TaqMan RT-qPCR assay for exon 3-4. All analyses are based on three experiments, with means ± SD. One representative gel per experiment is shown. Comparisons were made against the vehicle control (DMSO). P values are for unpaired two-sided Student's T-test. The source data are provided in the Source Data file.

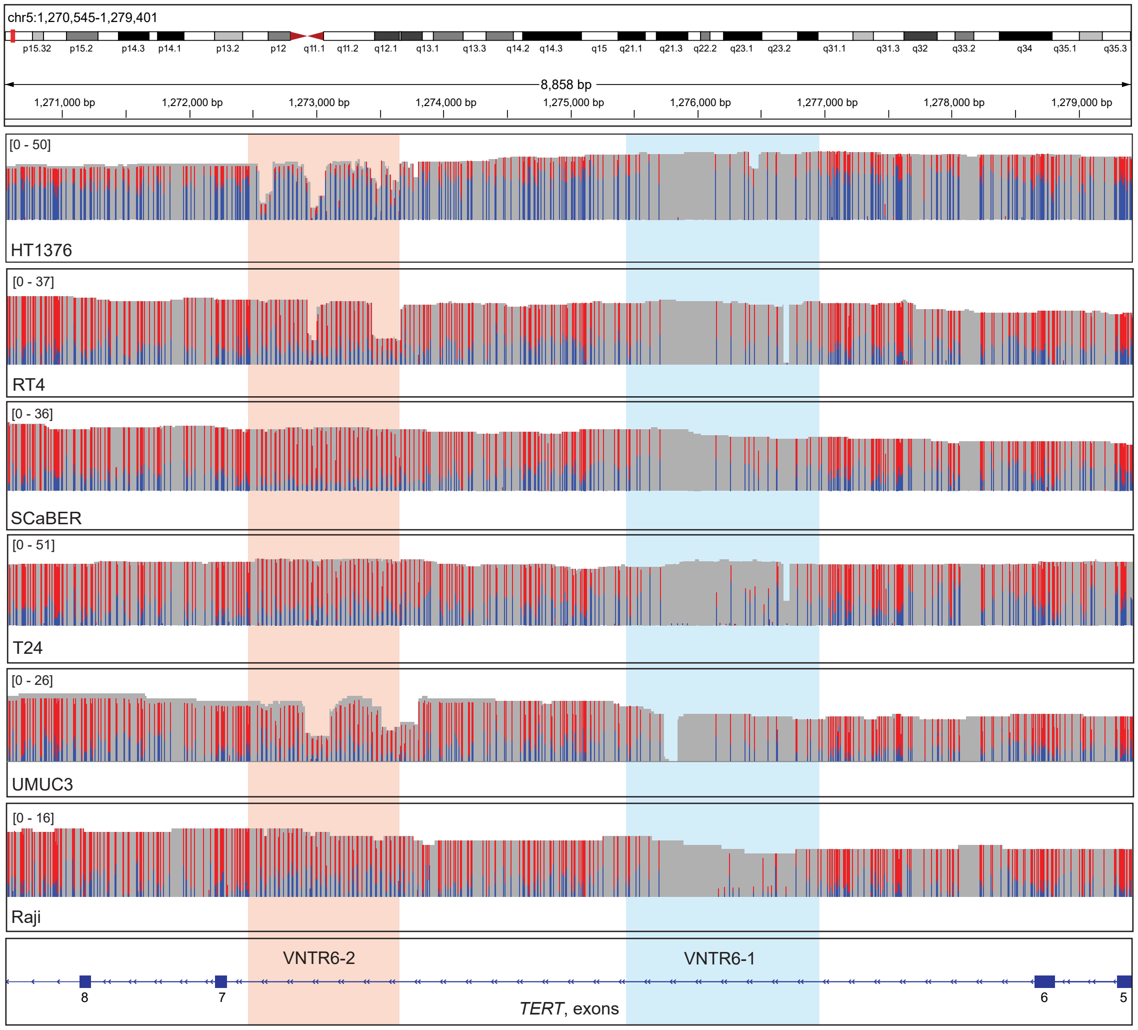

Supplementary Figure 9. DNA methylation profile of *TERT* VNTR6-1 region in PacBio WGS alignments.

IGV plots of PacBio WGS alignments in bladder cancer cell lines HT1376, RT4, T24, SCaBER, UMUC3, and a Burkitt lymphoma cell line Raji. CpG sites are marked based on the probabilities of 5-methylcytosine (5mC) modifications as low (< 50%, blue) and high (≥ 50%, red); gray areas - no CpG sites. The areas of VNTR6-1 and VNTR6-2 within *TERT* intron 6 are highlighted, with the VNTR6-1 region being largely devoid of CpG sites. The details are provided in the Source Data file.

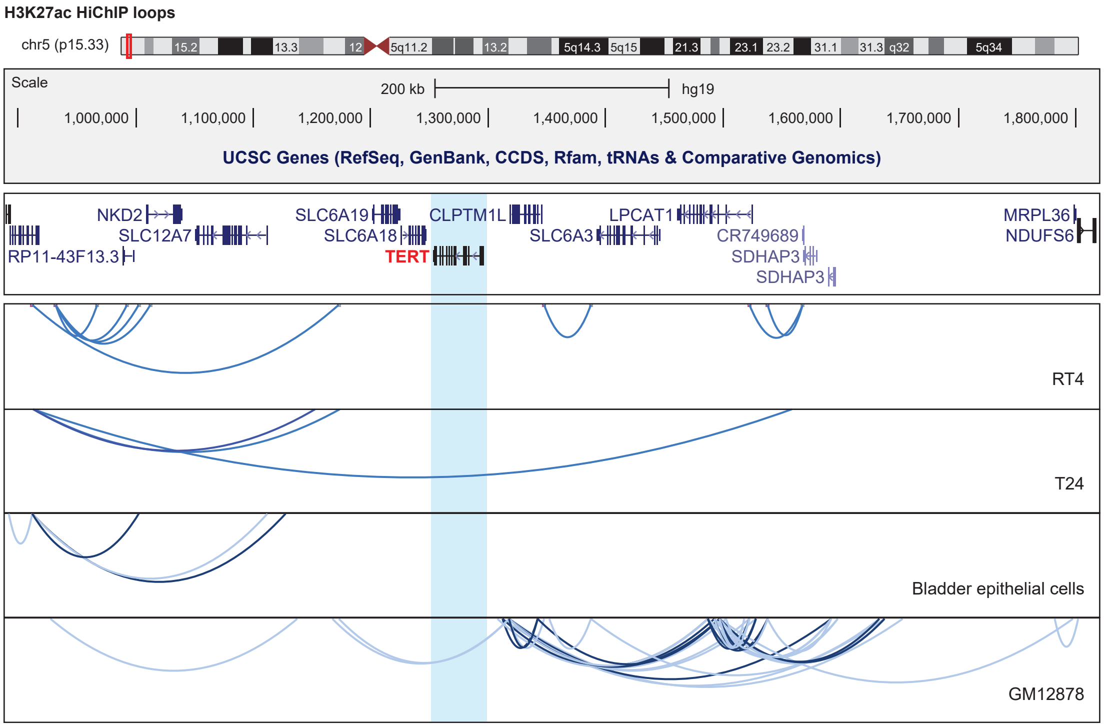

Supplementary Figure 10. Lack of long-range chromatin interactions within the *TERT* region.

H3K27Ac HiChIP loop tracks in bladder cancer cell lines RT4 and T24 (combined biological triplicates for each cell line), normal bladder epithelial cells, and lymphoblastoid cell line GM12878. The darker-colored loops correspond to a higher confidence based on statistical significance. The details are provided in the Source Data file.

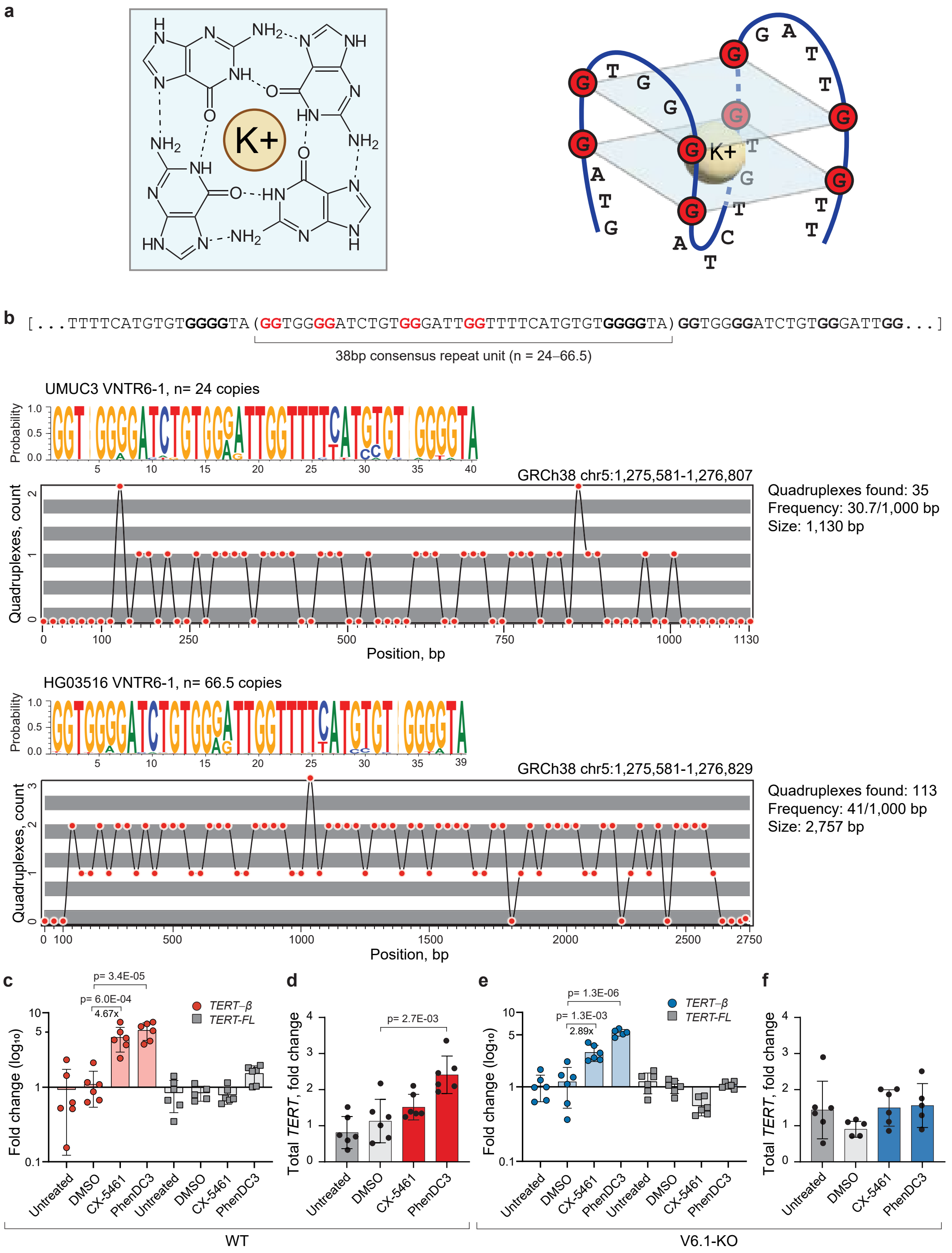

Supplementary Figure 11. Analysis of G-quadruplexes (G4) formed by VNTR6-1.

a, Schematic top-view of a G4 structure characterized by hydrogen-bonded guanines (G) stabilized by a central cation (K+); a model of the G4-forming folding of VNTR6-1, and VNTR6-1 consensus sequence (38-bp repeat unit) with marked guanines (G) contributing to one G4 unit shown in the model. **b,** Sequence logos of the VNTR6-1 repeat unit and prediction of G4s across the VNTR6-1 region using G4Hunter, with each dot representing the predicted number of G4s at a corresponding position. Results are presented for one allele per sample for both the shortest VNTR6-1 version (UMUC3 cells with 24 repeat copies) and the longest VNTR6-1 version (HG03516 cells with 66.5 repeat copies) observed in human populations. **c**, **e**, Isoform-specific TaqMan RT-qPCR analysis of *TERT-FL* and *TERT-β* following treatment with G4-stabilizing ligands CX-5461 and PhenDC3 for 72 hours. **d**, **f**, Total *TERT* expression measured by TaqMan RT-qPCR assay targeting the exon 3-4 junction. All analyses are based on one of three representative experiments. Comparisons were made against the vehicle control (DMSO). Data is presented as means ± SD. P values were calculated for unpaired two-sided Student's T-test. The source data are provided in the Source Data file.

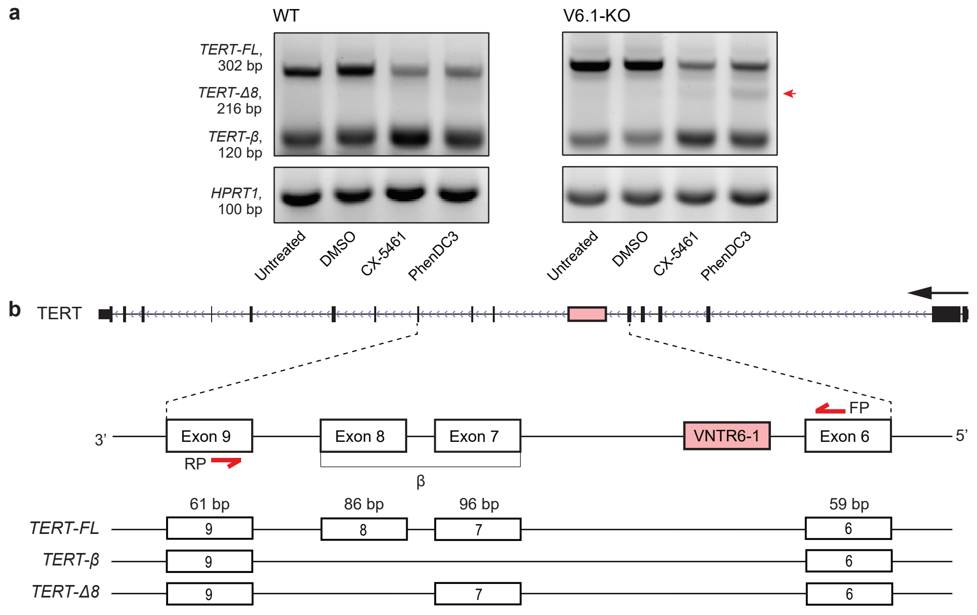

Supplementary Figure 12. An alternative isoform *TERT-Δ8*.

a, Agarose gel electrophoresis of SYBR Green qRT-PCR products from UMUC3 cells revealing a novel *TERT* isoform lacking exon 8 in cells exposed to G4 stabilizing ligands (images from **Figure 2c, d**). A representative gel from three independent experiments is shown. All distinct PCR bands were gel-extracted, cloned into the TOPO-TA vector, and Sanger sequenced in a single experiment, confirming the identity of all PCR products and revealing that the unexpected 216 bp PCR band results from exon 8 skipping. **b**, The schematic illustrates the alternative splicing of *TERT* exons 6-9, with red arrows denoting RT-PCR primers. Exons 7 and 8 are expected to be included or skipped together; however, in the presence of G4 stabilizing ligands, exon 8 was spliced both with and without exon 7. Skipping of exon 8 (86 bp) results in a frameshift and premature transcript termination within exon 10 in a pattern similar to *TERT-β.* Both *TERT-β* and *TERT*-Δ8 produce telomerase-nonfunctional *TERT* and are likely to be eliminated by NMD.

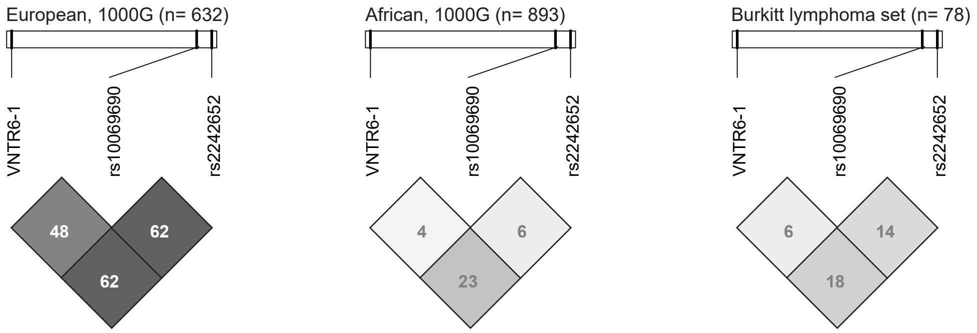

Supplementary Figure 13. Linkage disequilibrium plots (LD, r^2) for individuals from 1000G-EUR and 1000G-AFR reference panels, and patients with Burkitt lymphoma (BL, 88% of African ancestry).

The values within cells are for pairwise r^2 between the markers, calculated for 1000G-EUR and 1000G-AFR controls and Burkitt lymphoma patients, with individual sample sizes for each group indicated in the figure. Details are provided in the Source Data file.

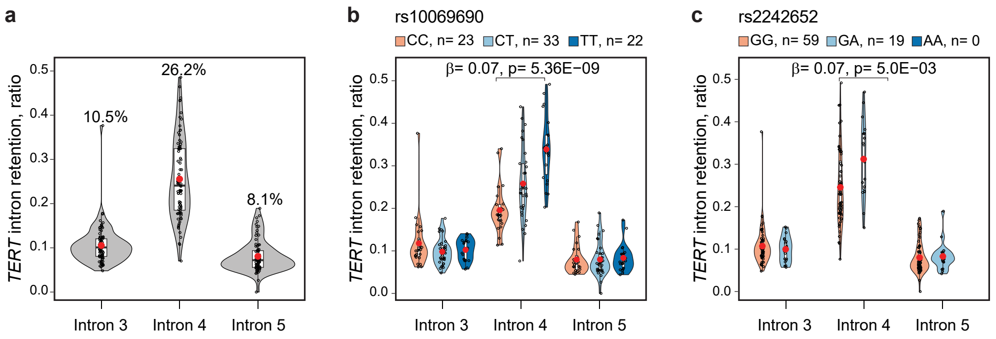

Supplementary Figure 14. Analysis of *TERT* intron retention in 78 Burkitt lymphoma (BL) tumors.

The mRNA intron retention ratios in BL tumors (n=78) were calculated for *TERT* introns 3, 4, and 5 on a scale from 0 (no retention) to 1 (full retention). **a**, Analysis in the full dataset of BL tumors and in relation to genotypes of GWAS signals **b**, rs10069690 and **c**, rs2242652, with individual sample sizes for each group indicated in the figure. The retention ratio is higher for *TERT* intron 4 than introns 3 and 5, and stronger associated with rs10069690 than rs2242652. The group means for retention ratios are shown as red dots and values above violin plots. Within each violin plot, the embedded box plots define the center line as the median, the whiskers as the minima and maxima, and the bounds as the 1st (25%) and 3rd (75%) quartiles of the distribution. P values and β-values are for linear regression models adjusting for sex and age. Details are provided in the Source Data file.

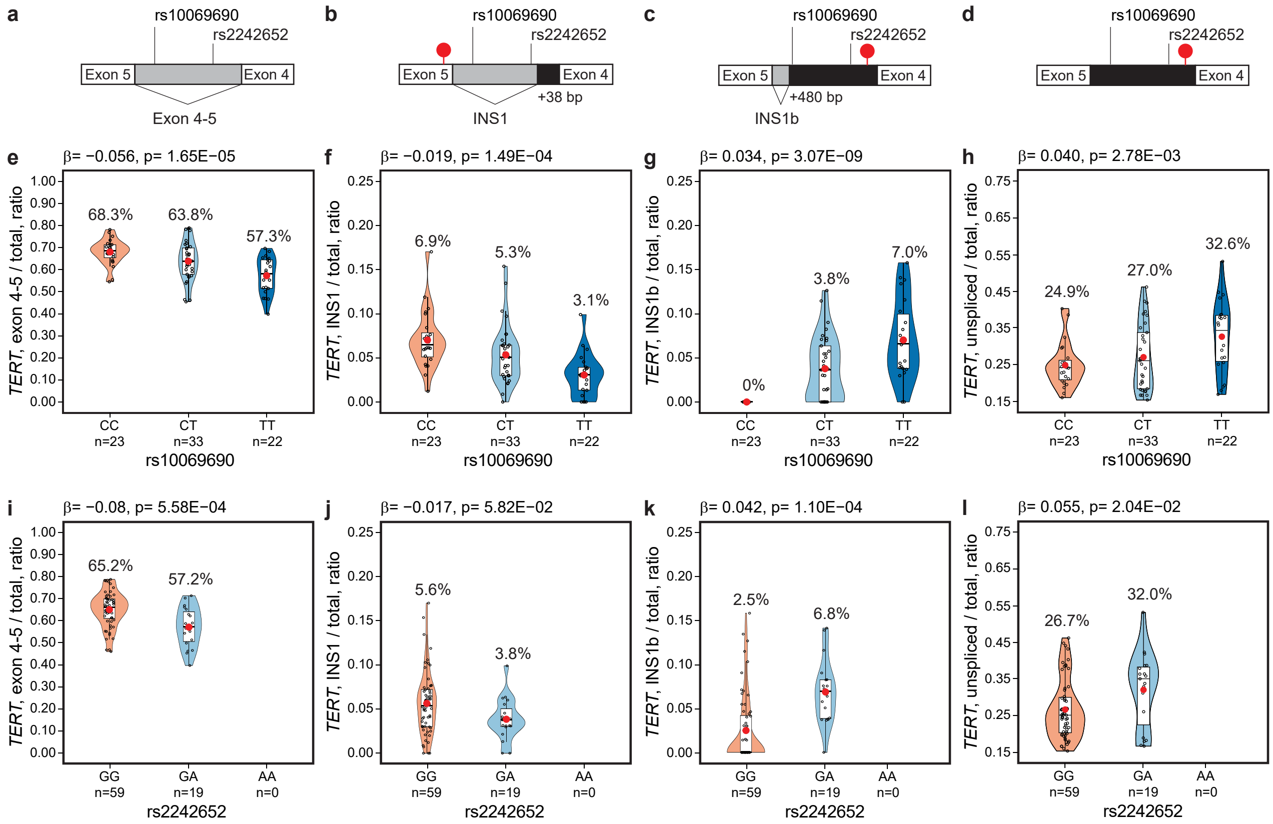

Supplementary Figure 15. Analysis of splicing events between *TERT* exons 4 and 5 in relation to GWAS signals rs10069690 and rs2242652 in 78 Burkitt lymphoma (BL) tumors.

Schematic of *TERT* intron 4 splicing or retention, which generates: **a**, canonical exon 4-5 splicing, **b**, *INS1* isoform with a stop codon in exon 5, **c**, *INS1b* isoform with a stop codon 48 bp downstream of exon 4, and **d**, unspliced intron 4 with a stop codon 48 bp downstream of exon 4. The ratios of reads and group means for BL tumors (n=78) at rs10069690 and rs2242652 and each splicing event relative to the sum of all read counts between exons 4 and 5, including intron retention reads for **e**, **i**, canonical exon 4-5 splicing; **f**, **j**, *INS1*-type splicing; **g**, **k**, *INS1b*-type splicing; **h**, **l**, unspliced intron 4. The group means are shown as red dots and values above violin plots. White boxes – exons, gray boxes – spliced introns, black boxes – retained introns, red lollipops – stop codons. Gene direction is shown from right to left. P values and β-values are for linear regression models adjusting for sex and age. Within each violin plot, the embedded box plots define the center line as the median, the whiskers as the minima and maxima, and the bounds as the 1st (25%) and 3rd (75%) quartiles of the distribution. Individual sample sizes for each group are indicated in the figure. Details are provided in the Source Data file.

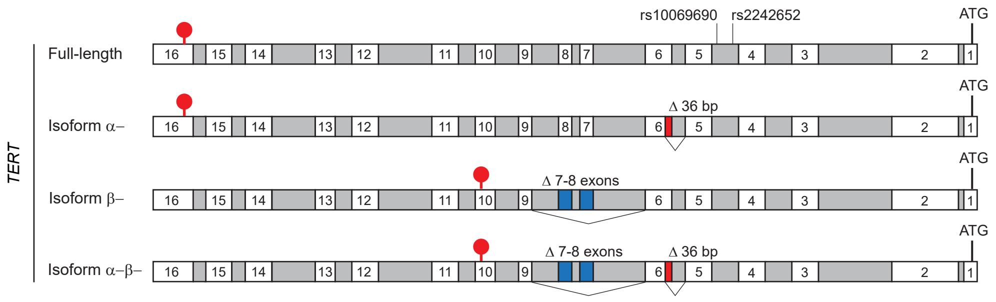

Supplementary Figure 16. Main *TERT* isoforms with alternative splicing within the area of GWAS signals rs10069690 and rs2242652.

White boxes – constitutive exons, red boxes – alternatively spliced exons, gray boxes – introns, red lollipops – stop codons. The direction of *TERT* exons is from right to left, corresponding to the minus strand as presented in the UCSC browser. "ATG" marks translation start codons.

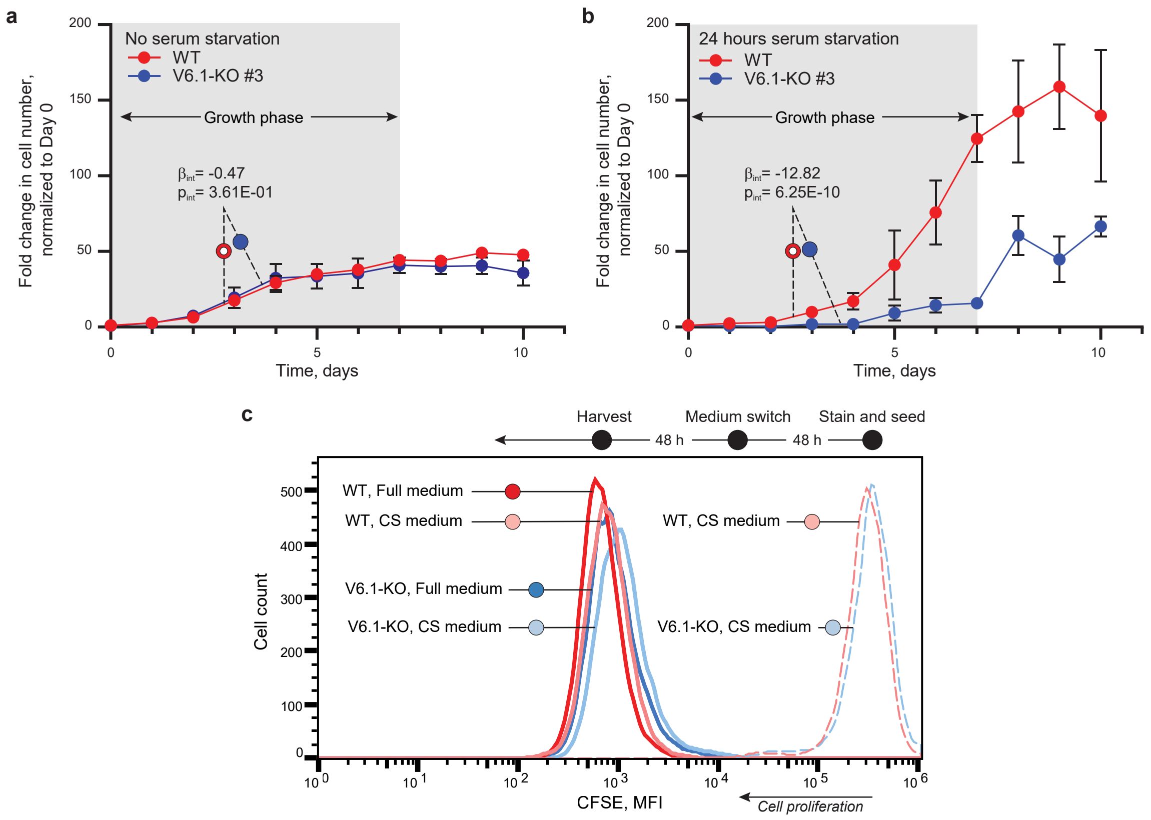

Supplementary Figure 17. VNTR6-1-associated responses to changes in culturing conditions in UMUC3 cells.

Cell proliferation was measured by label-free cell counting on the Lionheart automated microscope. The cells were cultured for 24 hours **a** without serum starvation or **b** with serum starvation, followed by switching to full medium for 10 additional days. The plots show average fold changes in counts of WT cells and one representative V6.1-KO clone (V6.1-KO clone #3, used in **Figure 4**). The graphs are based on one experiment with means ± SEM across four biological replicates. Statistical significance and β-values for differences in the cell index during the visually determined growth phase (gray highlighting from 0 to 7 days) were calculated using linear mixed-effects interaction models based on four replicates per sample. The reference sample is labeled with a dotted circle; β_{int} represents the change in growth rates between experimental groups. **c,** Experimental design for proliferation analysis based on depletion of CFSE stain, for which fluorescence intensity (CFSE, MFI) is decreased by half with every cell doubling. The protocol comprises three steps: 1) Stain and seed - WT and V6.1-KO cells were stained with CFSE and seeded on the same day in CS medium; starting mean fluorescence intensities (MFI) of CFSE ($CFSE_{start}$) were measured by flow cytometry; 2) Medium switch - 48 hours post-seeding, the CS medium was replaced with either fresh CS medium or full medium; and 3) Harvest and analysis – 48 hours after medium switch, cells were harvested and final MFI of CFSE ($CFSE_{final}$) was measured by flow cytometry. The overlay plot represents data from three independent experiments, showcasing results for WT cells and one representative V6.1-KO clone (V6.1-KO clone #3, used in **Figure 4**). Data values and gating strategy are provided in the Source Data file.

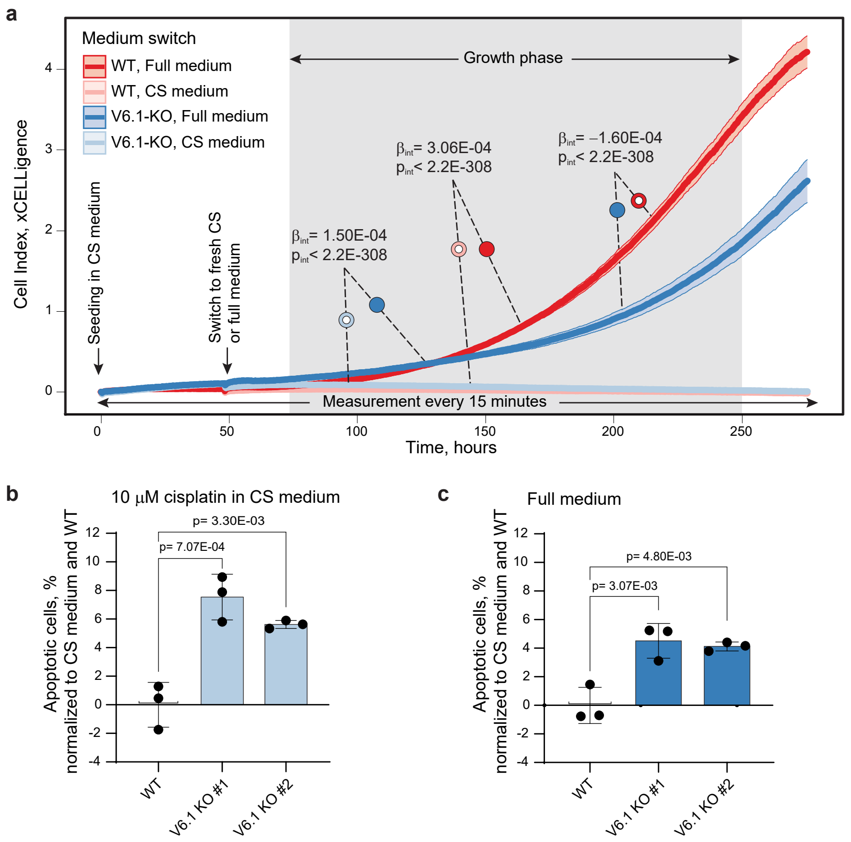

Supplementary Figure 18. Effects of *TERT* VNTR6-1 knockout on proliferation and apoptosis in A549 cells.

a, Analysis of real-time increase in cell counts (cell index) measured with xCELLigence over 275 hours in A549 cells. The WT cells and V6.1-KO clone #2 (starred samples in **Supplementary Data 9**) were cultured in CS medium for 48 hours, followed by the switch to fresh CS or full medium for 10 more days. Proliferation rates in response to culturing conditions were significantly decreased in V6.1-KO compared to WT cells. The plot is based on one representative experiment of three performed, plotting means ± SEM across five biological replicates. Statistical significance and β-values for differences in the cell index during the visually determined growth phase (gray highlighting between 72 and 250 hours) were calculated using linear mixed-effects interaction models. The reference sample is labeled with a dotted circle; β_{int} represents the change in growth rates between experimental groups. **b, c,** Quantification of apoptosis in WT and V6.1-KO A549 cells cultured for 48 hours **b** with 10 μM cisplatin in CS medium or **c** in full medium, followed by Annexin V/PI staining to determine the percentage of apoptotic cells. Data are presented as means ± SD for three biological replicates (normalized to values of CS media and the WT groups), P-values in graphs are for one-way ANOVA using Dunnett's test. Data values are provided in the Source Data file.

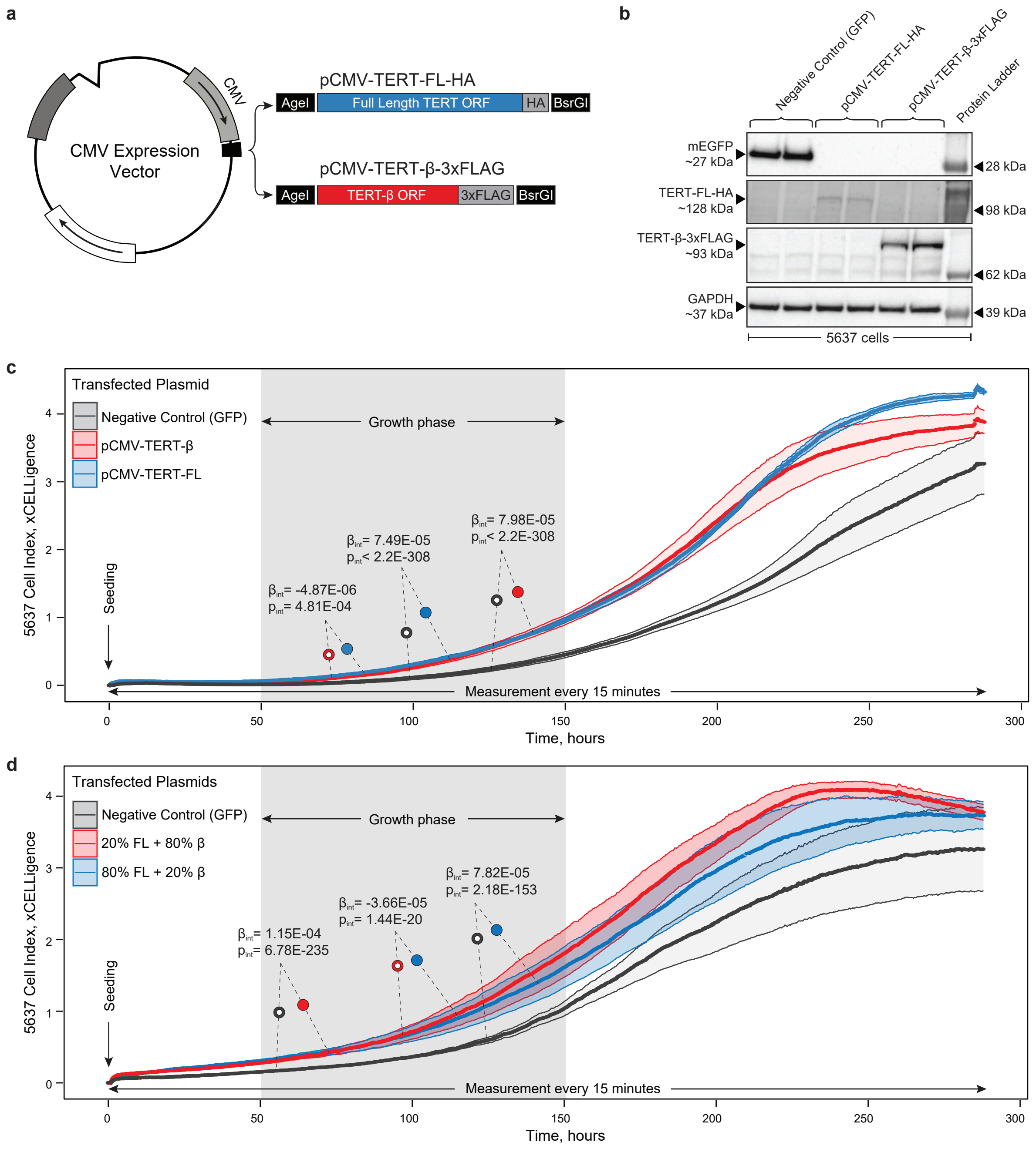

Supplementary Figure 19. Effects of *TERT* isoforms overexpression.

a, Cloning diagram for *TERT* isoforms. **b,** Western blot confirming the antibody quality and protein levels of TERT-FL-HA and TERT-β-3xFLAG overexpressed in the bladder cancer cell line (5637) with two biological replicates each. BCA-normalized samples were used across four separate membranes, which were probed with primary antibodies, including the loading control GAPDH, and processed in parallel. Unprocessed western blot images are provided in Source Data. **c, d,** Analysis of real-time increase in cell counts (cell index) measured with xCELLigence over 288 hours in 5637 cells following **c**, single transfection of expression plasmids for *TERT* isoforms or **d**, co-transfection of *TERT* isoforms in either 80:20% or 20:80% ratios of transfected plasmids (TERT-FL-HA:TERT-β-3xFLAG). The plot is based on one of three representative experiments, plotting means ± SEM across **c**, five or **d**, four biological replicates. Statistical significance and β-values are for differences in cell index during the visually determined growth phase (gray highlight between 50 and 150 hours) and were calculated using linear mixed-effects interaction models based on **c**, five or **d**, four biological replicates. The reference sample is labeled with a dotted circle; β_{int} represents the change in growth rates between experimental groups. Data values are provided in the Source Data file.

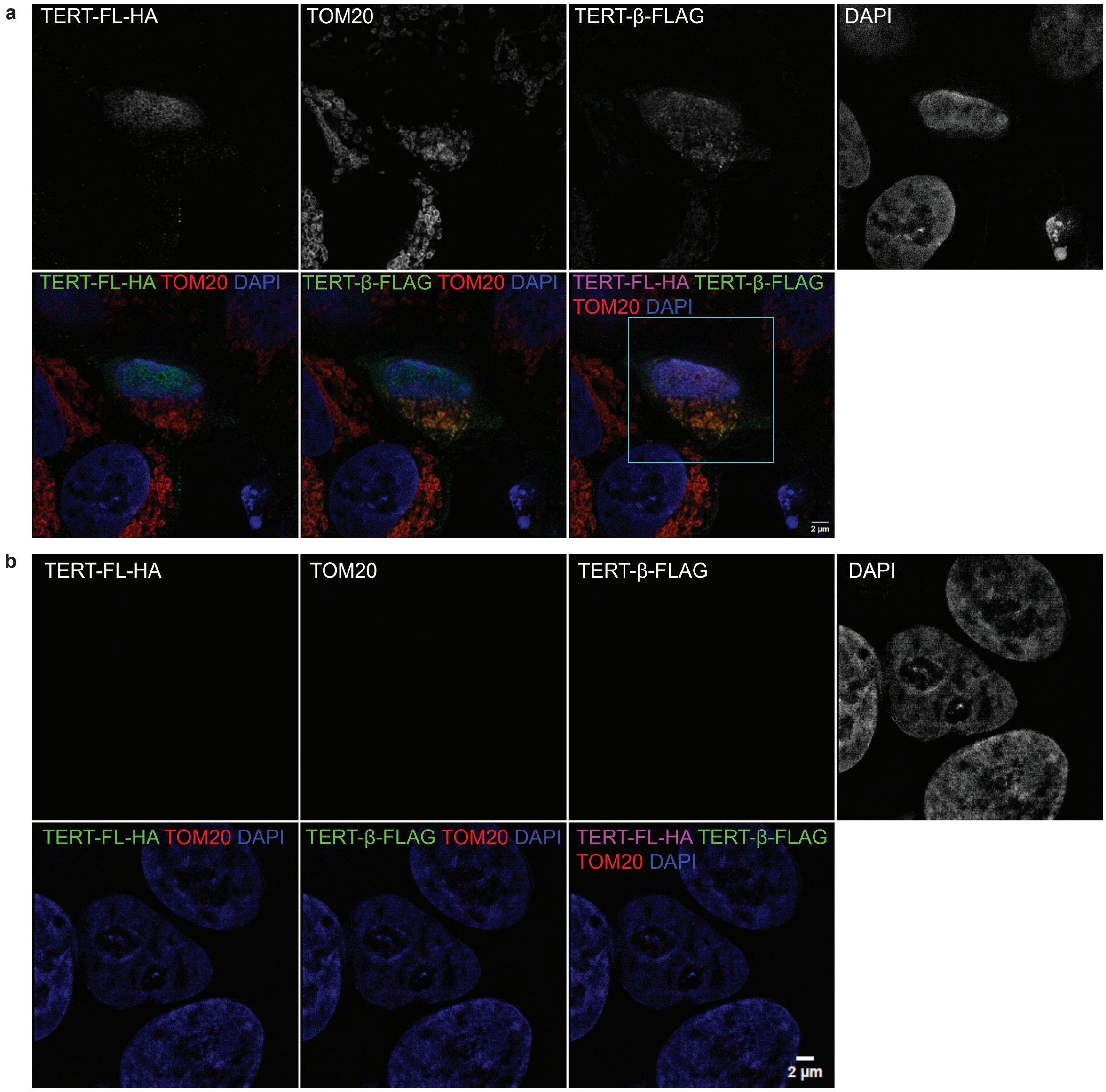

Supplementary Figure 20. Cellular localization of the TERT-FL and TERT-β protein isoforms.

Structured illumination microscopy (SIM) images of the TERT-FL and TERT-β protein isoforms transiently overexpressed in the A549 lung cancer cell line. Cells were co-transfected with TERT-FL-HA and TERT-β-FLAG expression constructs at a 50:50% ratio and stained with corresponding antibodies. For individual channels, staining is shown as black/white images for better contrast. On tri-color merged panels, green – FLAG (TERT-β) or HA (TERT-FL), blue – DAPI (nuclei). On the quad-color merged panel, purple – HA (TERT-FL), green – FLAG (TERT-β), red – TOM20 (mitochondria), blue – DAPI (nuclei). **a**, Zoomed-out view of transfected cells stained with anti-FLAG and anti-TOM20 primary antibodies, as well as respective secondary antibodies AlexaFluor647-/AlexaFluor488- (pseudocolored green) and AlexaFluor555- (pseudocolored red). The orange color indicates the mitochondrial colocalization of TOM20 with TERT-β, not seen for TERT-FL. The region in the cyan box is zoomed-in in **Figure 5a. b**, View of negative controls: similarly transfected cells but omitting primary antibodies and stained only with secondary antibodies, confirming that the detected signals are not from autofluorescence. Images were captured with a 63x/1.4 NA objective. Scale bars=2 μm. Images shown are representative of two independent experiments.

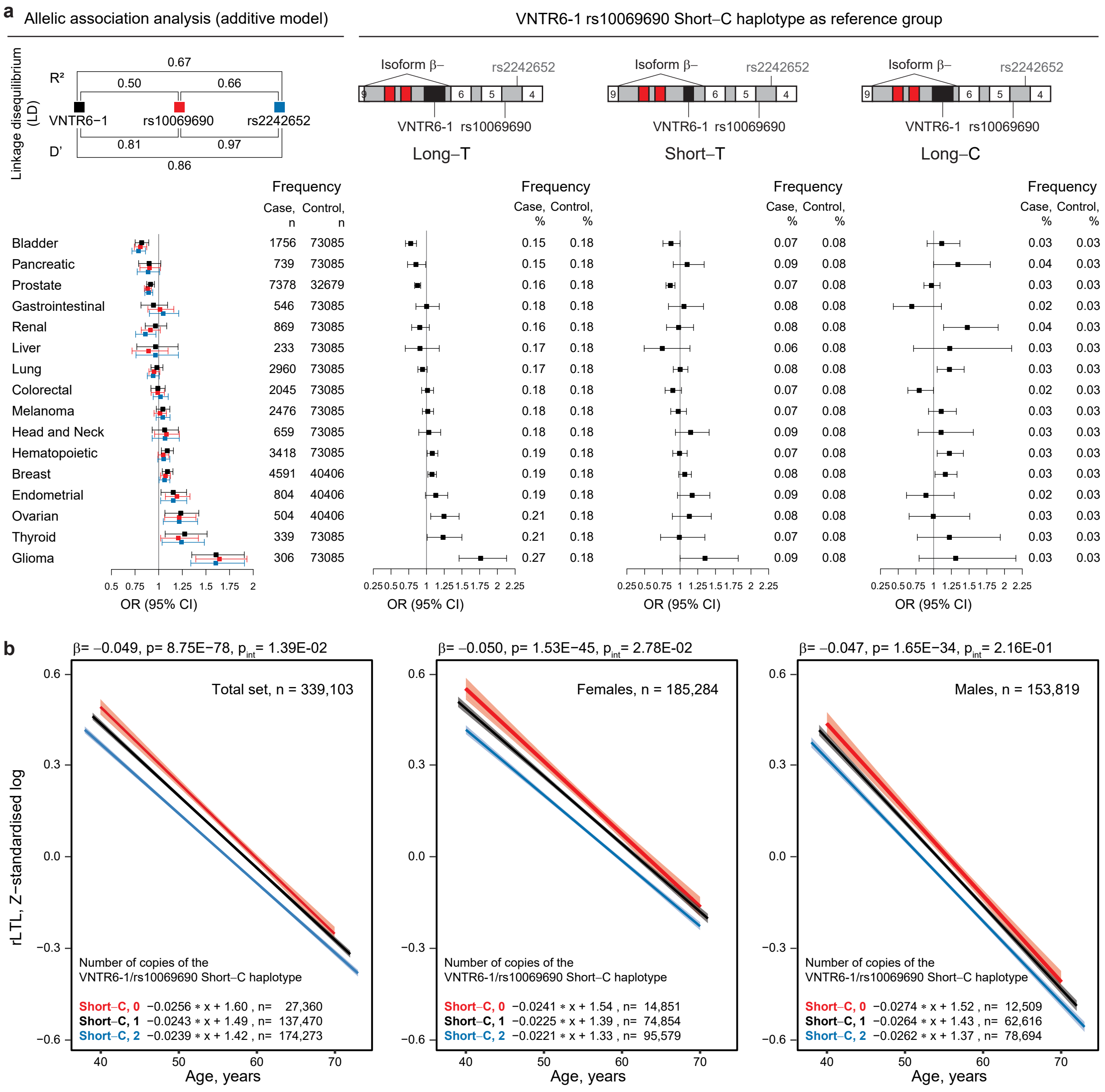

Supplementary Figure 21. Association analysis of cancer risk in PLCO and relative leukocyte telomere length (rLTL) in UKB cancer-free individuals.

a, Evaluation of cancer risk in the PLCO dataset (n=102,708) associated with *TERT* VNTR6-1-Long, rs10069690-T and rs2242652-A alleles and haplotypes of the composite marker (VNTR6-1/rs10069690) compared to the Short-C haplotype). Odds Ratios (OR) with 95% confidence intervals (CI) were calculated and plotted comparing patients with indicated cancers to a common group of cancer-free controls, using logistic regression analysis and an additive genetic model, adjusting for sex and age. **b**, Evaluation of the relationship in UKB cancer-free individuals (n=339,103) between rLTL and the VNTR6-1/rs10069690 marker. P values and β-values were derived from linear regression models, adjusted for sex, age, and smoking status, and for age and smoking status in sex-specific analyses (**Supplementary Data 16**). P_{int} are for interaction between genotypes and age groups. The graphs display regression lines with 95% confidence intervals and regression equations. The analysis shows a decrease in rLTLs with more copies of the Short-C haplotype. Individual sample sizes for each group are indicated in the figure. The details are provided in the Source Data file.

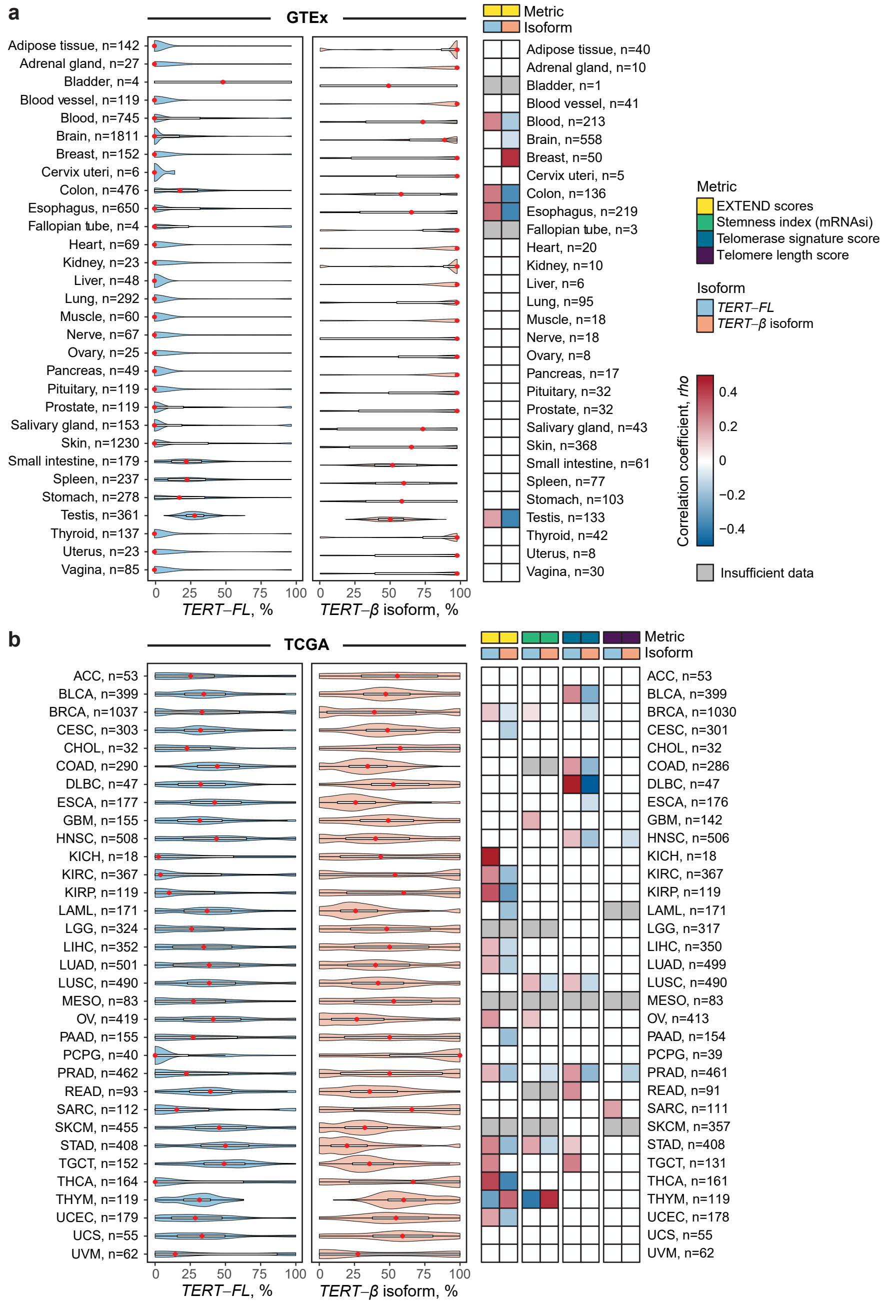

Supplementary Figure 22. Analysis of isoform-level *TERT* expression in relation to telomerase-associated metrics in GTEx and TCGA.

Isoform-level *TERT* expression (full-length (FL) and β-isoform) as a percentage of total *TERT* expression analyzed as transcripts per million (TPMs) in **a**, normal tissue samples in GTEx and **b**, tumors in TCGA. The mean expression for each tissue and tumor type is represented by a red dot within each violin plot. Spearman rank correlation coefficients (rho) between *TERT* expression (full-length and β-isoform) and various telomerase-associated metrics in **a**, GTEx and **b**, TCGA. Only statistically significant correlations are shown in color, gray – insufficient data for analysis; n indicates the number of samples analyzed. TCGA data is for one sample per individual, GTEx data combined samples from each tissue type, for some tissues (brain, skin) this includes several samples per individual. Within each violin plot, the embedded box plots define the center line as the median, the whiskers as the minima and maxima, and the bounds as the 1st (25%) and 3rd (75%) quartiles of the distribution. Full results are presented in **Supplementary Data 14**. The source data are provided in the Source Data file.

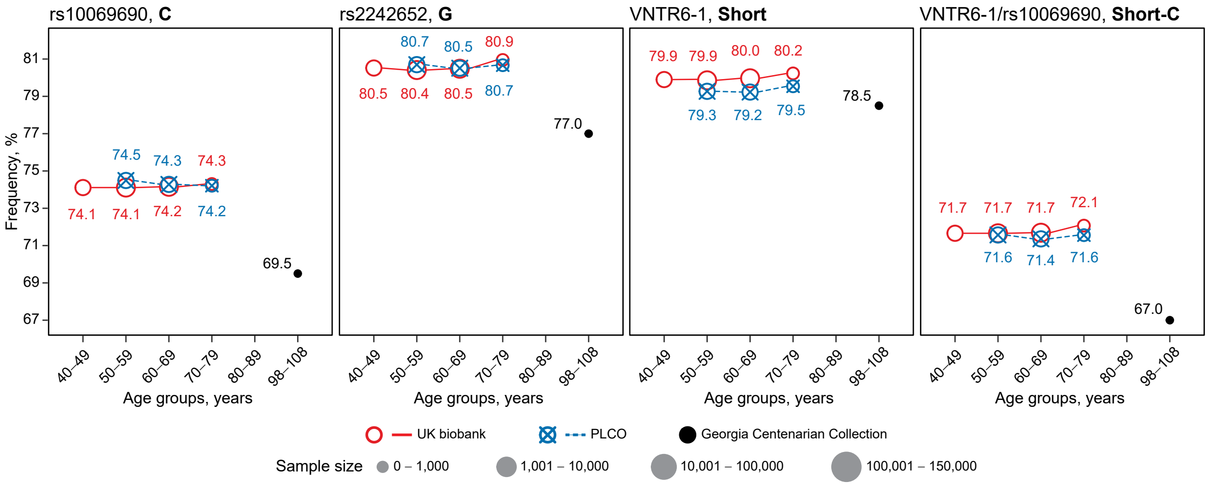

Supplementary Figure 23. Age-dependent frequencies of *TERT* alleles in individuals of European ancestry.

The distribution of the derived human-specific alleles rs10069690-C and VNTR6-1-Short and ancestral rs2242652-G allele in cancer-free individuals from the UK biobank (n=351,629) and PLCO (n=73,084), and in the Georgia Centenarian Collection (n=100). Dot sizes correspond to the sample numbers in each age group. Alleles rs10069690-C and VNTR6-1-Short and Short-C haplotype are associated with higher total *TERT* expression and a lower ratio of alternative non-telomerase producing isoforms *INS1b* (rs10069690-C) and *TERT-β* (Short).

In this set of centenarians of European ancestry, the decrease in frequencies of derived alleles is unlikely due to potential population stratification, as the frequency of the ancestral rs2242652-G allele is the lowest in the populations of European ancestry (1000G-EUR, 79%), while higher in other populations (1000G-AFR, 88%, 1000G-EAS, 85%, 1000G-AMR, 85%, 1000G-SAS, 82%). A potential admixture with other populations would result in higher, not lower rs2242652-G allele frequency observed in centenarians of European ancestry (77.0%). The details are provided in the Source Data file.

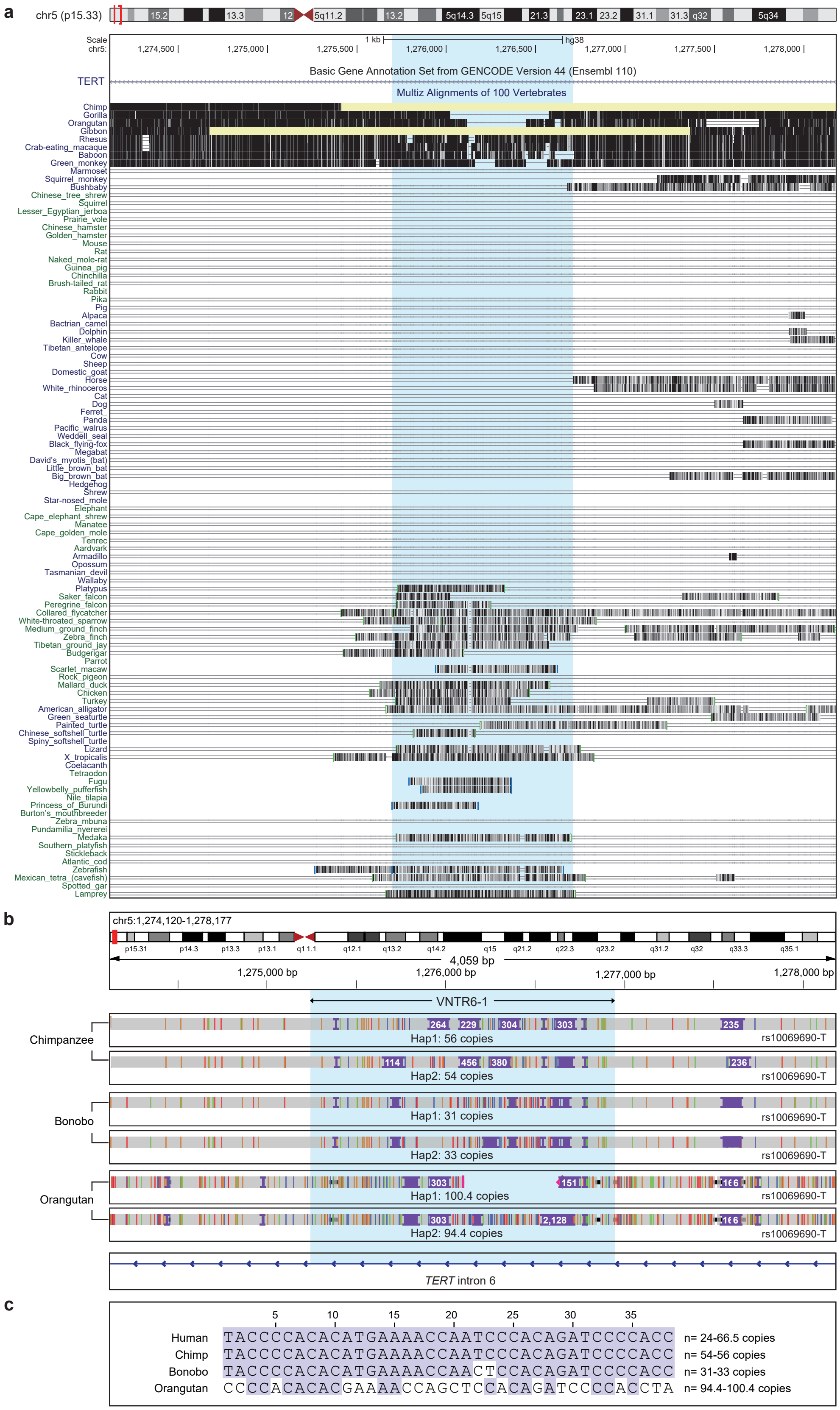

Supplementary Figure 24. *TERT* VNTR6-1 region across vertebrate species.

a, Multiple sequence alignment in 100 vertebrate species (source: UCSC Genome Browser). **b,** IGV plots of primate Telomere-to-Telomere assemblies aligned to the human genome (GRCh38). Shown are the VNTR6-1 region (blue highlight) with the number of repeat copies per haplotype and rs10069690 alleles within the same haplotype. **c,** Comparison of the VNTR6-1 consensus repeat unit (38-bp) between humans and primates. The details are provided in the Source Data file.

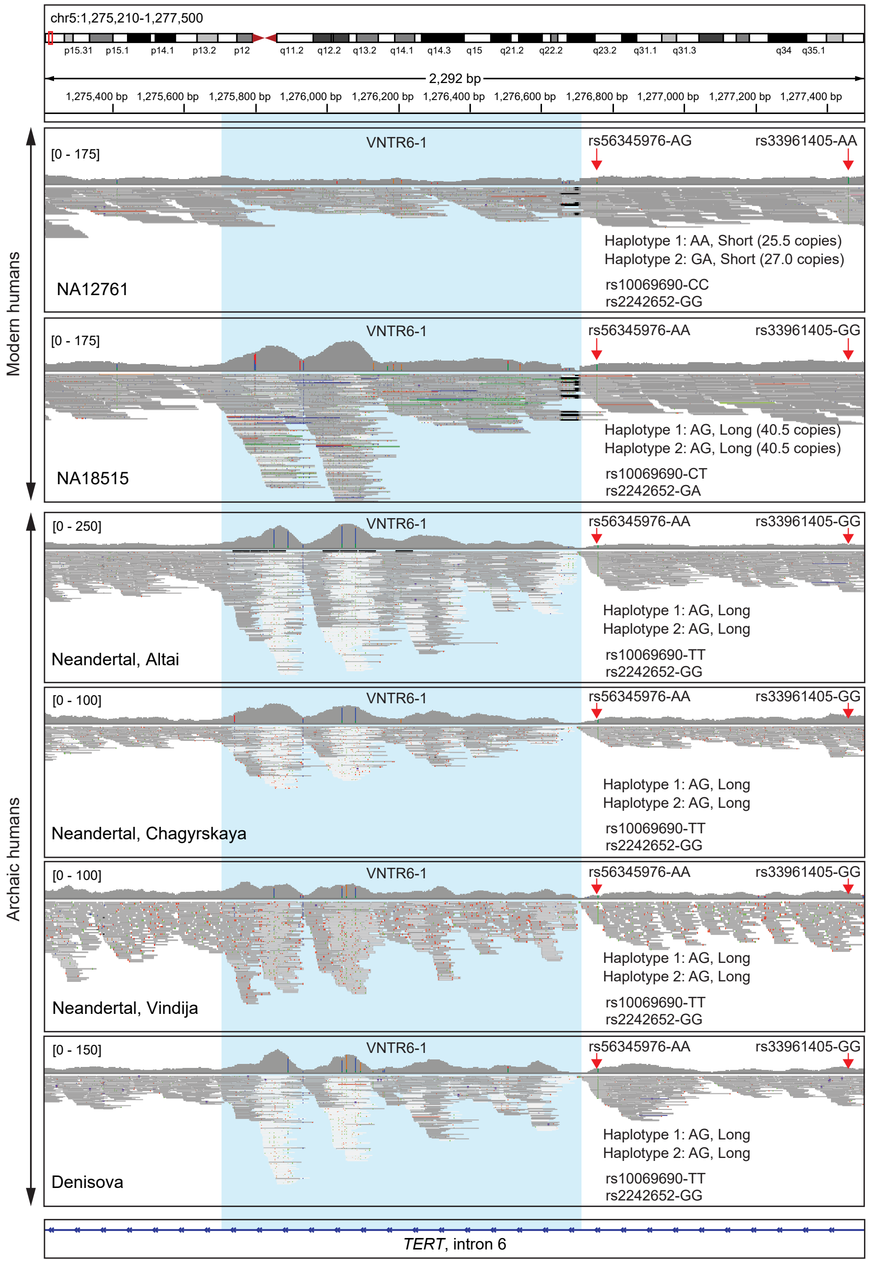

Supplementary Figure 25. *TERT* VNTR6-1 profiles in short-read WGS alignments in modern and archaic humans.

IGV plots of short-read WGS (Illumina, 30x coverage) illustrating VNTR6-1 (blue highlight) and SNPs rs56345976 and rs33961405 in modern humans (NA12761, Short/Short, and NA18515, Long/Long) and archaic humans (Neandertal and Denisova, all VNTR6-1-Long/Long genotypes). The genotypes of the multi-cancer GWAS leads rs2242652 and rs10069690 are shown for corresponding samples. The details are provided in the Source Data file.
