# Supplementary material for: Genetic regulation of TERT splicing affects cancer risk by altering cellular longevity and replicative potential
Source: Nat Commun. 2025 Feb 16;16:1676. doi: 10.1038/s41467-025-56947-y (PMC11830802; doi:10.1038/s41467-025-56947-y)
Supplement: Supplementary file 2 — Description of Additional Supplementary Information [file 41467_2025_56947_MOESM2_ESM.docx]

**Description of Additional Supplementary Files**

File Name: **Supplementary Data 1**

Description: Analysis of VNTR6-1 and VNTR6-2 within TERT intron 6 in relation to cancer-related GWAS leads in long-read assemblies.

File Name: **Supplementary Data 2**

Description: VNTR6-1 characterization based on HPRC long-read assemblies or targeted PacBio sequencing in various datasets.

File Name: **Supplementary Data 3**

Description: Genotypes of phased SNPs and VNTR6-1 based on short-read WGS in 1000G dataset.

File Name: **Supplementary Data 4**

Description: Machine learning random forest analysis of SNP-based prediction of VNTR6-1 groups in 3,201 individuals of diverse ancestries from the 1000G.

File Name: **Supplementary Data 5**

Description: Linkage Disequilibrium (LD) analysis between VNTR6-1 and SNPs with MAF>5% in the GRCh38 chr5:1,100,000-1,500,000 region across all populations in the 1000 Genomes Project.

File Name: **Supplementary Data 6**

Description: Distribution of VNTR6-1 alleles in relation to haplotypes of rs56345976 and rs33961405.

File Name: **Supplementary Data 7**

Description: Association of rs10069690 and rs2242652 with TERT expression in GTEx and TCGA.

File Name: **Supplementary Data 8**

Description: TERT expression patterns in 78 BL tumors in relation to rs10069690, rs2242652, VNTR6-1, and haplotypes of VNTR6-1-rs10069690.

File Name: **Supplementary Data 9**

Description: xCELLigence real-time monitoring of cell growth in V6.1-KO and WT cells.

File Name: **Supplementary Data 10**

Description: RNA-seq differential gene expression analysis comparing UMUC3-V6.1 KO with UMUC3 WT (reference).

File Name: **Supplementary Data 11**

Description: Gene Ontology (GO) pathway analysis comparing UMUC3-V6.1 KO with UMUC3 WT (reference).

File Name: **Supplementary Data 12**

Description: xCELLigence real-time cell growth monitoring of the bladder cancer cell line 5637 transfected with TERT-FL and TERT-β expression constructs.

File Name: **Supplementary Data 13**

Description: Association analyses with cancer risk in PLCO dataset of individuals of European ancestry.

File Name: **Supplementary Data 14**

Description: Relative expression of TERT-FL and beta isoforms in GTEx and TCGA.

File Name: **Supplementary Data 15**

Description: Correlation of TERT isoform expression with telomerase-related metrics in GTEx and TCGA.

File Name: **Supplementary Data 16**

Description: Association of relative leukocyte telomere length (rLTL) in cancer-free individuals from the UK Biobank.

File Name: **Supplementary Data 17**

Description: Association of flow FISH telomere length in cancer-free individuals from Transplant Outcomes of Aplastic Anemia (TOAA) cohort.

File Name: **Supplementary Data 18**

Description: Distribution of VNTR6.1, rs10069690 and rs2242652 in the 1000G super-populations, PLCO, UK BioBank and Georgia Centenarian Collection.

File Name: **Supplementary Data 19**

Description: Sequences of DNA and RNA oligos used in the study.
