# Supplementary material for: Genetic regulation of TERT splicing affects cancer risk by altering cellular longevity and replicative potential
Source: Nat Commun. 2025 Feb 16;16:1676. doi: 10.1038/s41467-025-56947-y (PMC11830802; doi:10.1038/s41467-025-56947-y)
Supplement: Supplementary file 22 — Reporting Summary [file 41467_2025_56947_MOESM22_ESM.pdf]

nature portfolio

Corresponding author(s): Ludmila Prokunina-Olsson

Last updated by author(s): Jan 17, 2025

Reporting Summary

Nature Portfolio wishes to improve the reproducibility of the work that we publish. This form provides structure for consistency and transparency in reporting. For further information on Nature Portfolio policies, see our Editorial Policies and the Editorial Policy Checklist.

Statistics

For all statistical analyses, confirm that the following items are present in the figure legend, table legend, main text, or Methods section.

n/a	Confirmed	
☐	☒	The exact sample size (*n*) for each experimental group/condition, given as a discrete number and unit of measurement
☐	☒	A statement on whether measurements were taken from distinct samples or whether the same sample was measured repeatedly
☐	☒	The statistical test(s) used AND whether they are one- or two-sided<br>*Only common tests should be described solely by name; describe more complex techniques in the Methods section.*
☐	☒	A description of all covariates tested
☐	☒	A description of any assumptions or corrections, such as tests of normality and adjustment for multiple comparisons
☐	☒	A full description of the statistical parameters including central tendency (e.g. means) or other basic estimates (e.g. regression coefficient) AND variation (e.g. standard deviation) or associated estimates of uncertainty (e.g. confidence intervals)
☐	☒	For null hypothesis testing, the test statistic (e.g. *F*, *t*, *r*) with confidence intervals, effect sizes, degrees of freedom and *P* value noted<br>*Give P values as exact values whenever suitable.*
☒	☐	For Bayesian analysis, information on the choice of priors and Markov chain Monte Carlo settings
☐	☒	For hierarchical and complex designs, identification of the appropriate level for tests and full reporting of outcomes
☐	☒	Estimates of effect sizes (e.g. Cohen's *d*, Pearson's *r*), indicating how they were calculated

Our web collection on statistics for biologists contains articles on many of the points above.

Software and code

Policy information about availability of computer code

Data collection	1. DNA samples for HapMap I, HapMap III, Human Pangenome Reference Consortium (HPRC), and Georgia Centenarian Collection were purchased from Coriell Institute for Medical Research. Deidentified tissue samples for bladder tumors and matching adjacent normal samples were purchased from Asterand Bioscience. Flow FISH telomere length samples were from donors of hematopoietic cell transplants from the Center for International Blood and Marrow Transplant Research (CIBMTR; https://cibmtr.org) biorepository.<br>2. Urinary bladder cell lines UMUC3 (CRL-1749), 5637 (HTB-9), HT1376 (CRL-1472), RT4 (HTB-2), T24 (HTB-4), and SCaBER (HTB-3), as well as a Burkitt lymphoma cell line Raji (CCL-86) and a lung cancer cell line A549 (CCL-185) were purchased from ATCC (Manassas).<br>3. Phased genome assemblies, RNA-seq, ATAC-seq, H3K27ac HiChIP-seq, and ChIP-seq datasets used in this study are detailed in Table S19, which includes the respective download links.<br>4. We used genetic data from the 1000 Genomes Project, GenomeArk, the Prostate, Lung, Colorectal, and Ovarian (PLCO) Cancer Screening Trial, the UK Biobank (UKB), Burkitt lymphoma tumors, The Cancer Genome Atlas (TCGA), and Genotype-Tissue Expression (GTEx), as detailed in Table S19.<br>5. Data generated by this project, including PacBio, HiChIP, WGS, and RNA-seq, is publicly available.
Data analysis	1. Genetic data was processed using SHAPEIT4 (v4.2.0) for phasing and IMPUTE2 (v2.3.2) for imputation.<br>2. RNA-seq BAM files were analyzed for splicing events using FeatureCounts (v2.0.6), isoform estimation with MISO (v0.5.4), and intron retention with IRFinder (v2.0.1).<br>3. Long-read sequences were processed to genotype SNPs using SAMtools with the mpileup flag (v1.17), and VNTRs were scored using Straglr (v1.4).<br>4. Short-read WGS data was used to score VNTR6-1 into Short and Long categories by applying a machine learning strategy with tidymodels based on the R package ‘glmnet’ (v4.1-7).

5. A random forest model applied using the R package 'randomForest' (v4.7-1.1) to identify variants predictive of VNTR6-1-Short/Long status across the 400 kb genomic region from the 1000G phased genetic variant data.
6. Logistic regression analyses for binary outcomes were conducted using the 'glm' function with a logit link, linear regression analyses were performed using the 'lm' function, and Spearman rank correlations were computed using the 'rcorr' function, all implemented in R (v4.3.0).
7. G4Hunter (https://bioinformatics.ibp.cz/#/) was used for predicting G4 structures
8. Longitudinal trajectories of cell growth data were analyzed using linear mixed-effects models implemented with the 'lme' function from the R nlme package (v3.1-153).
9. Our RNA-seq data from experimental cell lines were processed as follows: alignment and transcript abundance estimation using Salmon (v0.14.1) and STAR (v2.1). Gene-level transcript abundances were estimated with 'lengthScaledTPM' in the 'tximport' R package (v1.28.0), followed by differential expression analysis using DESeq2 (v1.40.2). Gene set enrichment analysis (GSEA) was performed using clusterProfiler (v4.8.3).
10. PacBio, Oxford Nanopore sequences, and FASTA sequences from GenomeArk were aligned to the GRCh38 reference genome using minimap2. PacBio DNA methylation sequences were aligned to GRCh38 using the default settings of the SMRT-Link workflow
11. Integrative Genomics Viewer version 2.8.9 (http://www.broadinstitute.org/igv) was used for WGS, RNA-seq, and PacBio DNA methylation visualization.
12. The UCSC Genome Browser was utilized for visualizing HiChIP interactions.
13. Unless indicated, analyses were performed with R Studio (v4.3.0), GraphPad Prism (v.10), FlowJo (v10.8.1), SnapGene (v7.0.1), and Fiji (v2.15.1).

For manuscripts utilizing custom algorithms or software that are central to the research but not yet described in published literature, software must be made available to editors and reviewers. We strongly encourage code deposition in a community repository (e.g. GitHub). See the Nature Portfolio guidelines for submitting code & software for further information.

Data

Policy information about availability of data

All manuscripts must include a data availability statement. This statement should provide the following information, where applicable:

- Accession codes, unique identifiers, or web links for publicly available datasets
- A description of any restrictions on data availability
- For clinical datasets or third party data, please ensure that the statement adheres to our policy

All sequencing data generated in this study (PacBio targeted sequencing, PacBio-WGS, HiChIP, and RNA-seq) have been deposited in the Sequence Read Archive (SRA) with the BioProject codes PRJNA1134698 (https://www.ncbi.nlm.nih.gov/bioproject/?term=PRJNA1134698) and PRJNA1134701 (https://www.ncbi.nlm.nih.gov/bioproject/PRJNA1134701). The publicly available datasets used in the study include RNAseq transcript expression data from TCGA, available on the UCSC Xena platform (https://toil.xenahubs.net) at https://toil-xena-hub.s3.us-east-1.amazonaws.com/download/tcga_rsem_isoform_tpm.gz; RNAseq transcript expression data from GTEx, available on the GTEx portal (https://www.gtexportal.org/) at https://storage.googleapis.com/adult-gtex/bulk-gex/v8/rna-seq/GTEx_Analysis_2017-06-05_v8_RSEMv1.3.0_transcript_tpm.gct.gz; haplotype-resolved Telomere-to-Telomere (T2T) assemblies of primates, available in the Genome Ark database (https://www.genomeark.org/) under IDs: mGorGor1, mPanPan1, mPanTro3, and mPonAbe1, accessed on October 3, 2013 (https://registry.opendata.aws/genomeark); BAM files of Neandertal and Denisova individuals, available in the Max Planck Institute for Evolutionary Anthropology (https://www.eva.mpg.de/index/) under IDs: Altai, Denisova, Vindija, and Chagyrskaya; FASTA long-read sequences files, available in the Human Pangenome Reference Consortium (https://humanpangenome.org/) at https://github.com/human-pangenomics/HPP_Year1_Data_Freeze_v1.0; HiChIP data, available in the Gene Expression Omnibus under accession code GSE18840180; the 1000 Genomes 30x on GRCh38 data, available in The International Genome Sample Resource (https://www.internationalgenome.org/) at https://www.internationalgenome.org/data-portal/data-collection/30x-grch38; and ChIP-seq data for G-quadruplexes, available in the Gene Expression Omnibus under accession code GSE6387473 and GSE11058274. The data from PLCO and UKB can be requested based on application and approval. The controlled access long-read sequencing data from the Center for Alzheimer's and Related Dementias (CARD) of the National Institute on Aging is available from dbGaP phs001300.v4.p1, and data for Burkitt Lymphoma Genome Sequencing Project (BLGSP) is available from dbGAP phs000527.v6.p2. The remaining data used in this article are available within the Article, Supplementary Information or Source data provided with this paper.

Research involving human participants, their data, or biological material

Policy information about studies with human participants or human data. See also policy information about sex, gender (identity/presentation), and sexual orientation and race, ethnicity and racism.

Reporting on sex and gender	All analyses were controlled for sex.
Reporting on race, ethnicity, or other socially relevant groupings	Analyses of TERT expression in Burkitt Lymphoma (BL) tumors were conducted on individuals of African ancestry because TERT expression is very high in these tumors and most indviduals with BL tumors are of African ancestry. Analyses of cancer risk and telomere length were restricted to individuals of European ancestry due to insufficient data in other ancestries.
Population characteristics	The datasets provide the total number of subjects, their distribution by sex, and details of age, including range and mean. All analyses were adjusted for age. Genotypic information was obtained from each dataset. Cancer and healthy control statuses were used for multi-cancer and telomere length association analyses.
Recruitment	Data for this study were retrieved from various datasets. Recruitment details are available in the study-specific references provided for each dataset.
Ethics oversight	The research presented in this paper complied with all relevant ethical regulations. The study used deidentified controlled access data from the Center for Alzheimer's and Related Dementias (CARD) of the National Institute on Aging (dbGaP phs001300.v4.p1), Burkitt Lymphoma Genome Sequencing Project (BLGSP, dbGaP phs000527.v6.p2), the Prostate, Lung, Colorectal and Ovarian (PLCO) Cancer Screening Trial (project #PLCO-957), UK Biobank (project #92005) and The Cancer Genome Atlas (TCGA, https://gdc.cancer.gov). The use of deidentified bladder tissue samples was approved by the NIH Office of Human Subjects Research (#4715). The use of deidentified samples from the Center for International Blood and Marrow Transplant Research biorepository (CIBMTR; https://cibmtr.org) was approved by the National Marrow Donor Program

Institutional Review Board. All study participants or their guardians provided informed consent for participation in the CIBMTR Research Database and Research Sample Repository Protocols (NCT01166009 and NCT00495300). Non-controlled access data were obtained from public resources – 1000 Genomes Project and GTEx.

Note that full information on the approval of the study protocol must also be provided in the manuscript.

Field-specific reporting

Please select the one below that is the best fit for your research. If you are not sure, read the appropriate sections before making your selection.

[x] Life sciences [ ] Behavioural & social sciences [ ] Ecological, evolutionary & environmental sciences

For a reference copy of the document with all sections, see nature.com/documents/nr-reporting-summary-flat.pdf

Life sciences study design

All studies must disclose on these points even when the disclosure is negative.

Sample size	Information on sample sizes for all data types used in this study is provided in the Supplementary tables and Methods
Data exclusions	Samples with missing genetic or covariate data were excluded from the analysis.
Replication	Cell line experiments included multiple replicates, whereas for human data analyses, permutations were used when the sample size was less than 100; otherwise, no replication was performed.
Randomization	This study did not include randomization.
Blinding	The visual scoring of VNTR6-1 in short-read WGS was performed blinded to other scoring methods.

Reporting for specific materials, systems and methods

We require information from authors about some types of materials, experimental systems and methods used in many studies. Here, indicate whether each material, system or method listed is relevant to your study. If you are not sure if a list item applies to your research, read the appropriate section before selecting a response.

Materials & experimental systems

n/a	Involved in the study	
[ ]	[x]	Antibodies
[ ]	[x]	Eukaryotic cell lines
[x]	[ ]	Palaeontology and archaeology
[x]	[ ]	Animals and other organisms
[x]	[ ]	Clinical data
[x]	[ ]	Dual use research of concern
[x]	[ ]	Plants

Methods

n/a	Involved in the study	
[x]	[ ]	ChIP-seq
[ ]	[x]	Flow cytometry
[x]	[ ]	MRI-based neuroimaging

Antibodies

Antibodies used

Target gene-conjugate (clone) | Cat. No. | Source | Target species | Host | Dilution
1. FLAG (M2) | F3165 | Sigma-Aldrich | Epitope Tag | Mouse | 1:1000 (WB), 1:400 (IF)
2. GAPDH (polyclonal) | ab9485 | Abcam | Human, mouse | Rabbit | 1:1000
3. GFP (polyclonal) | A-11122 | Invitrogen (ThermoFisher) | Epitope Tag | Rabbit | 1:1000
4. HA (polyclonal) | NB600-362 | Novus | Epitope Tag | Goat | 1:1000 (WB), 1:400 (IF)
5. TOM20 (polyclonal) | 11802-1-AP | Proteintech | Human, Mouse, Rat, Chicken | Rabbit | 1:1000
6. anti-Rabbit-HRP | 7074 | Cell Signaling | Rabbit | Goat | 1:5000
7. anti-Rabbit-AlexaFluor555 | A31572 | ThermoFisher | Rabbit | Donkey | 1:500
8. anti-Mouse-HRP | 7076 | Cell Signaling | Mouse | Horse | 1:5000
9. anti-Mouse-AlexaFluor488 | A21202 | ThermoFisher | Mouse | Donkey | 1:500
10. anti-Goat-HRP | sc-2304 | Santa Cruz | Goat | Donkey | 1:5000
11. anti-Goat-AlexaFluor647 | A32849 | ThermoFisher | Goat | Donkey | 1:500

Validation

For FLAG, GFP, and HA (epitope) antibodies: transfections of single plasmids expressing tagged proteins showed positive staining of respective target tags and negative staining for non-target tags. GAPDH ab9485 was validated by manufacturer for WB in HeLa, A432, and A549 cells. TOM20 11802-1-AP was validated by manufacturer for WB in HEK-293, HeLa, and HepG2; and for IF in HUVEC and HepG2 cells.

Eukaryotic cell lines

Policy information about cell lines and Sex and Gender in Research

Cell line source(s)	All the cell lines were purchased from the American Type Culture Collection (ATCC).
Authentication	AmpFlSTR Identifiler Plus Kit (Thermo Fisher Scientific)
Mycoplasma contamination	All cell lines in the laboratory are regularly tested for mycoplasma contamination using the MycoAlert Mycoplasma Detection kit (Lonza). Cell lines tested negative when compared to positive control.
Commonly misidentified lines (See ICLAC register)	No commonly misidentified cell lines were used.

Plants

Seed stocks	*Report on the source of all seed stocks or other plant material used. If applicable, state the seed stock centre and catalogue number. If plant specimens were collected from the field, describe the collection location, date and sampling procedures.*
Novel plant genotypes	*Describe the methods by which all novel plant genotypes were produced. This includes those generated by transgenic approaches, gene editing, chemical/radiation-based mutagenesis and hybridization. For transgenic lines, describe the transformation method, the number of independent lines analyzed and the generation upon which experiments were performed. For gene-edited lines, describe the editor used, the endogenous sequence targeted for editing, the targeting guide RNA sequence (if applicable) and how the editor was applied.*
Authentication	*Describe any authentication procedures for each seed stock used or novel genotype generated. Describe any experiments used to assess the effect of a mutation and, where applicable, how potential secondary effects (e.g. second site T-DNA insertions, mosiacism, off-target gene editing) were examined.*

Flow Cytometry

Plots

Confirm that:

- [x] The axis labels state the marker and fluorochrome used (e.g. CD4-FITC).
- [x] The axis scales are clearly visible. Include numbers along axes only for bottom left plot of group (a 'group' is an analysis of identical markers).
- [x] All plots are contour plots with outliers or pseudocolor plots.
- [x] A numerical value for number of cells or percentage (with statistics) is provided.

Methodology

Sample preparation	Cells lines (UMUC3 urinary bladder and A549 lung carcinoma) were purchased and cultured per ATCC's recommended protocols. Cells were stained and analyzed as described in Methods.
Instrument	Attune NxT (with Cytkick)
Software	Attune Cytometric Software for acquisition, FlowJo v10.8.1 for analysis
Cell population abundance	In general, 10,000 or greater events were collected on Single Cell gates, unless cell death induced by cisplatin left fewer total events available (then maximum number of events were captured, which were no lower than 2500)
Gating strategy	Graphical figures with gating strategy are provided in Source Data 1. FSC/SSC: gate on Cells, 2. FSC-A/FSC-H: gate on Single Cells, 3. Fluorescent channel of interest, CFSE histogram: geometric mean calculated; Annexin V-FITC/PI: gates between unstained and stained for each color

- [x] Tick this box to confirm that a figure exemplifying the gating strategy is provided in the Supplementary Information.
